# FDX1-mediated cuproptosis promotes cholestatic liver injury exacerbated by taurocholic acid-enhanced copper accumulation

**DOI:** 10.1038/s41420-025-02861-7

**Published:** 2026-01-09

**Authors:** Yujun Guo, Min Yang, Shengbo Sun, Zhaohua Zhong, Wenjun Lu, Ze’nan Zhang, Meili Fan, Aodan Zhang, Tingting Zhang, Yang Wu, Zhou Li, Zuwei Liu, Qijun Sun, Zhaozhu Li, Qingbo Cui

**Affiliations:** 1 Department of Pediatric Surgery, The Sixth Affiliated Hospital of Harbin Medical University, Harbin, China; 2 Department of Microbiology, Harbin Medical University, Harbin, China; 3 Laboratory of Systems Immunology, Institute of Basic Medical Science, Westlake Institute for Advanced Study, Hangzhou, China; 4 Department of Pediatric Surgery, Women and Children’s Hospital, School of Medicine, Xiamen University, Xiamen, China; 5 Psychology and Health Management Center, Harbin Medical University, Harbin, China; 6 Department of Pediatric Surgery, The Second Affiliated Hospital of Harbin Medical University, Harbin, China; 7 Department of Hematology, The Second Affiliated Hospital of Harbin Medical University, Harbin, China

**Keywords:** Cell death, Hepatotoxicity

## Abstract

Cholestatic liver injury, characterized by direct exposure of hepatocytes to retained bile components with elevated concentrations, represents a common manifestation of various hepatobiliary disorders with persistent threats to long-term patient survival despite existing therapies. As the primary route for copper elimination, cholestasis raises questions about the role of copper in cholestatic liver injury and its specific molecular mechanisms. Our single-center retrospective study revealed elevated serum copper levels in subjects with increased gamma-glutamyl transferase compared to controls. Single-cell sequencing of biliary atresia (BA) patients’ cholestatic liver specimens demonstrated downregulation of FDX1, a key cuproptosis marker, in BA hepatocytes. Bile duct-ligated rats under high-copper diets exhibited accelerated liver injury, attenuated by copper chelator tetrathiomolybdate (TTM). In vitro, copper chloride/elesclomol-induced DLAT monomer reduction and oligomerization alongside impaired lipoylation. Given the special coexistence of copper overload and accumulated bile components within the hepatic microenvironment, notably, we found that taurocholic acid potentiated hepatic copper accumulation under cholestatic conditions. Mechanistically, transcriptomic analysis implicated smoothened signaling inhibition in cuproptosis progression, with smoothened agonist (SAG) restoring DLAT expression and cellular viability. Interestingly, FDX1 overexpression enhanced cuproptosis resistance of hepatocytes through DLAT monomer stabilization and LIAS-mediated lipoylation. Cholestasis-induced copper overload drives liver injury via taurocholic acid-exacerbated and FDX1-mediated cuproptosis. Our findings propose TTM and SAG as therapeutic candidates and reveal complex FDX1 regulatory roles, suggesting novel approach for managing cholestatic liver injury.

## Introduction

Cholestasis is the predominant commonality in multiple hepatobiliary diseases, including biliary atresia (BA), congenital biliary dilatation, Alagille syndrome, etc [1–3]. Due to disruption of bile flow and increased osmotic pressure, biliary components obligately accumulate in intrahepatic biliary system and hepatic parenchyma through disrupted bile canaliculi [4]. Overdose of multiple bile compositions directly causes hepatotoxicity, which leads to liver injury manifested by evaluated serum alanine aminotransferase (ALT) and gamma-glutamyl transpeptidase (GGT). Untreated cholestasis may progress to hepatic fibrosis, ultimately culminating in cirrhosis [5–7].

Copper serves as an essential catalytic cofactor for enzymes in a wide spectrum of biological processes [8], the level of which is tightly regulated at around 70 μM in eukaryotic cells [9]. Dysregulation of this process induces copper-mediated cytotoxicity, as evidenced by Wilson’s disease, a congenital autosomal recessive disorder characterized by pathogenic variants in the *ATP7B* copper transporter gene [10, 11]. Given that excess copper is eliminated by biliary secretion physically [8], in the case of cholestasis, disrupted biliary drainage leads to acquired biliary copper accumulation in hepatobiliary system [12]. Excess of copper, together with other bile composition, causes hepatotoxicity and liver fibrosis subsequently [13].

Cuproptosis was discovered recently as a novel regulated cell death, during which copper overload triggers the whole programmed procedure [8, 14, 15]. This copper-dependent form of cell death is characterized by a loss of Fe-S proteins and aggregation of lipoylated mitochondrial enzymes relevant to tricarboxylic acid cycle, which are important in mitochondrial respiration and cellular energy metabolism [16]. Ferredoxin 1 (FDX1), an upstream regulator of protein lipoylation, serves as the key regulatory factor in cuproptosis [17–19]. *FDX1* knockout by CRISPR/Cas9 conferred resistance to cuproptosis, whereas FDX1 was downregulated in copper/ionophore-induced cell death. We speculated that the regulatory role of FDX1 is complex, which warrants further study.

The main characterization of cholestasis is the accumulation of bile acids in the liver and serum, with dramatically increased levels [20–22]. Studies have reported that direct exposure of hepatocytes to bile acids induces cell death and liver injury mainly by hepatocellular apoptosis [23–25]. However, the concentration of individual bile acids in vivo may not actually reach the level to induce cell death of hepatocytes in vitro [26–28]. Thus, further studies regarding the exact mechanism of cholestatic-induced hepatocyte death should focus on the complex interaction of hepatocytes with the mixture of bile components.

Taurocholic acid (TCA), a primary conjugated bile acid, is one of the prominently increased bile acids in cholestatic liver [26, 27]. TCA uptake by sodium^+^/taurocholate co-transporting polypeptide-activated hepatic stellate cells and promoted liver fibrosis [29]. In a prospective clinical study, TCA was reported to be positively associated with liver cancer risk and fatal liver disease risk [30]. Pharmacological inhibition of intestinal bile acid absorption ameliorated cholestatic liver injury and fibrosis by reducing TCA concentration [31]. Due to the spatiotemporal coexistence of accumulated TCA and excess copper in cholestasis, the cross-talk between TCA-induced cytotoxicity and copper-initiated cuproptosis needs further investigation.

In the present study, we aim to clarify the role of FDX1-mediated cuproptosis in cholestatic liver injury. Further, the effect of TCA on copper-initiated cuproptosis has also been explored. Given that cuproptosis is primarily regulated through post-translational mechanisms, we investigated transcriptional alterations in cuproptotic hepatocytes. Taken together, our study elucidated the role of cuproptosis in cholestatic liver injury and the underlying regulatory network, providing insights for therapeutic strategies.

## Results

### Elevated serum copper levels in children with cholestasis

**Fig. 1:**
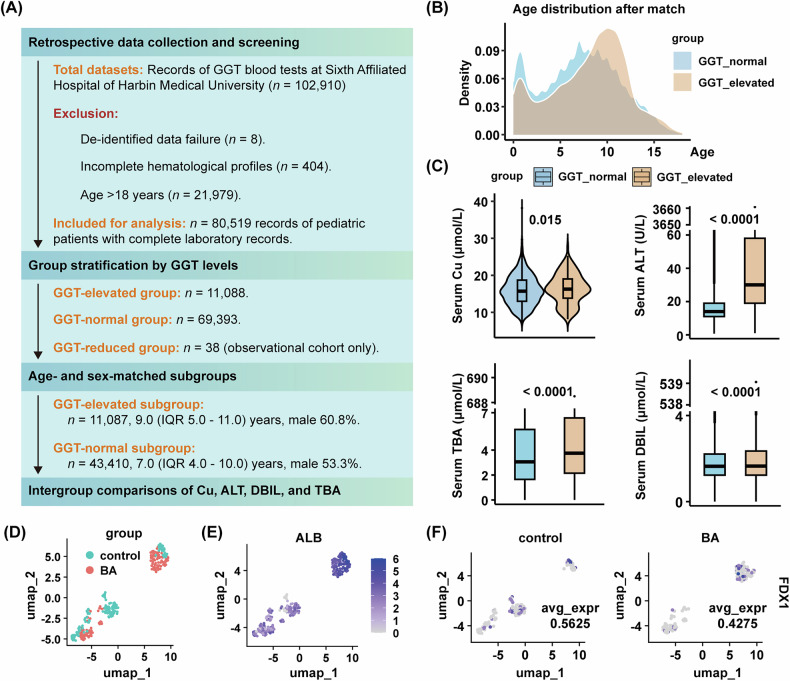
Elevated levels of serum copper and cholestatic bio-markers, and decreased FDX1 expression of hepatocytes in children with cholestasis. **A** Flowchart of retrospective clinical study design. Laboratory test results and relevant clinical data were collected from the Sixth Affiliated Hospital of Harbin Medical University between August 2022 and March 2025. **B** Density plot of age distribution between groups after matching for age and gender distribution. **C** Violin plot and boxplots displaying serum levels of Cu, ALT, TBA, and DBIL between GGT-normal group and GGT-evaluated group. The y-axis was truncated (range 60 to 3650 for ALT, range 7 to 688 for TBA, and range 4 to 538 for DBIL) to resolve compression artifacts caused by extreme values. Full-range visualization and statistical details of outliers are provided in Supplementary Fig. 1D. **D**, **E**, **F** UMAP plots illustrating hepatocytes derived from scRNA-seq of BA livers and matched control livers without cholestasis (GEO: GSE176189; *n* = 3). Sample origin (**D**), ALB gene expression (**E**), and FDX1 expression split by sample origin (**F**) of hepatocytes. Data are median (IQR). Mann–Whitney test (**C**). BA biliary atresia, GGT gamma-glutamyl transpeptidase, ALT alanine aminotransferase, TBA total bile acids, DBIL direct bilirubin, UMAP Uniform manifold approximation and projection, scRNA-seq single-cell RNA sequencing.

To investigate whether copper-related mechanisms mediate cholestatic liver injury, we retrospectively analyzed de-identified laboratory data from *n* = 80,519 pediatric hospital attendees undergoing diagnostic procedures (age ≤ 18 years). Given the high sensitivity of GGT to cholestasis, the attendees were stratified into three groups based on GGT levels: GGT-elevated group (GGT > upper limit of normal), GGT-normal group, and GGT-reduced group (GGT < lower limit of normal). After matching for age and gender distribution, GGT-elevated group (*n* = 11,087, 9.0 [IQR 5.0–11.0] years, male 60.8%) and GGT-normal group (*n* = 43,410, 7.0 [IQR 4.0–10.0] years, male 53.3%) were included for subsequent analysis (Fig. 1A, B, Supplementary Fig. 1A, B).

Biochemical profiling revealed higher serum copper levels in the GGT-elevated group compared to controls (16.3 [IQR 13.8–19.0] μmol/L vs. 15.7 [IQR 13.0–18.7] μmol/L, *p* = 0.015). This copper dysregulation paralleled increased ALT (30.0 [IQR 19–58] U/L vs. 14.0 [IQR 11–19] U/L, *p* < 0.0001), direct bilirubin (DBIL; 1.65 [IQR 1.23–2.35] μmol/L vs. 1.64 [IQR 1.23–2.21] μmol/L, *p* < 0.0001), and total bile acids (TBA; 3.75 [IQR 2.15–6.58] U/L vs. 3.06 [IQR 1.65–5.65] U/L, *p* < 0.0001; Fig. 1C). These findings are consistent with the clinical features of cholestatic liver injury and suggest copper overload as a novel characteristic feature of cholestatic liver injury.

### ScRNA-seq analysis reveals decreased FDX1 expression in hepatocytes of pediatric cholestasis patients

Given that BA is one of the most prominent cholestatic liver diseases, we integrated scRNA-seq hepatic profiles from three BA patients with cholestasis and three choledochal cyst controls without cholestasis (Supplementary Fig. 1E, F). Hepatocyte clusters were identified through albumin (ALB) expression profiling, with cluster 19 (ALB-high-expressing population) selected for subsequent analyses (Supplementary Fig. 1G). Uniform Manifold Approximation and Projection (UMAP) visualization demonstrated distinct spatial segregation between BA-derived and control-derived hepatocytes while maintaining consistent ALB expression patterns across groups (Fig. 1D, E). Quantitative analysis revealed diminished expression of FDX1, a pivotal copper-induced cell death regulator, which is decreased in cuproptosis, in BA hepatocytes compared to controls (Fig. 1F), suggesting the potential involvement of cuproptosis in cholestatic liver injury pathogenesis.

### Obstructive cholestasis induces dysregulated copper homeostasis with concomitant liver injury and cuproptotic changes in rats

**Fig. 2:**
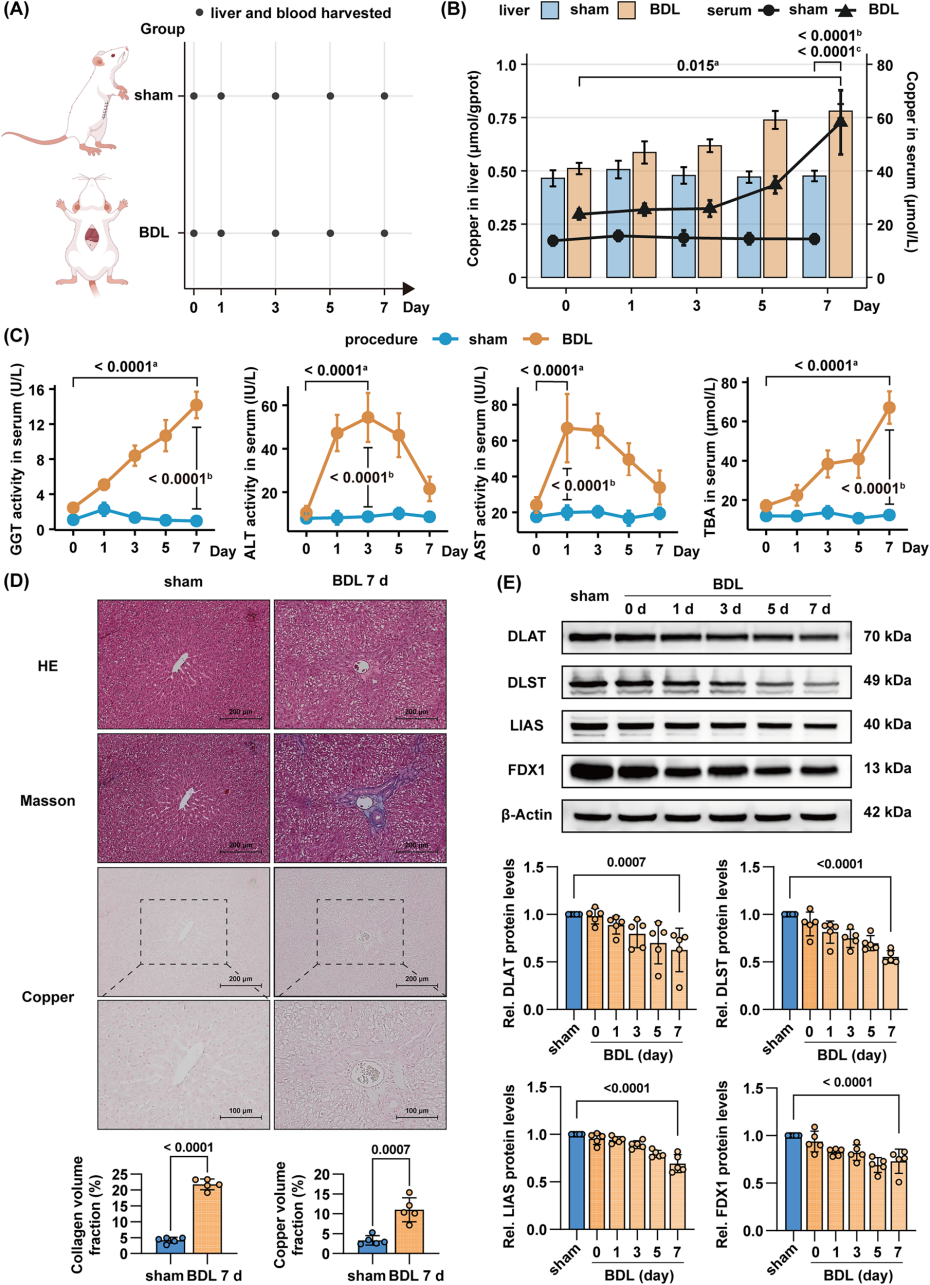
Obstructive cholestasis induces dysregulated copper homeostasis with concomitant liver injury and cuproptotic changes in rats. **A** Schematic diagram of animal experiments. Liver and blood of rats were harvested at 0, 1, 3, 5, and 7 days post-operation (“Day 0” refers to tissue collection performed 2–6 h after the completion of modeling surgery, when the animals had recovered from anesthesia and achieved stable vital signs). Dynamic changes in serum and liver copper levels (**B**, *n* = 6), serum GGT, ALT, and AST activities (**C**, *n* = 6), and serum TBA levels (**C**, *n* = 6). **D** Representative images of serial liver sections stained with H&E, Masson’s trichrome for collagen deposition (blue), and dithiooxamide for copper salt deposits (green granules, *n* = 5). Liver tissue of the sham group was harvested on day 7. Scale bar = 200 μm. Bottom: fold change in collagen volume fraction and copper volume fraction relative to sham-day 7 group. **E** Western blot of DLAT, DLST, LIAS and FDX1 in liver tissue of rats (*n* = 5). Liver tissue of the sham group was harvested on day 7. Bottom: fold change in DLAT, DLST, LIAS and FDX1 expression relative to sham-day 7 group. Data are mean ± SD. Two-way ANOVA with Tukey’s correction (**B**, **C**), unpaired Student’s *t* test (**D**), and one-way ANOVA with Šídák’s correction (**E**). ^a^ Comparing serum values between BDL groups at designated single time point. ^b^ Comparing serum values between sham and BDL groups at designated single time point. ^c^ Comparing liver values between sham-day 7 and BDL-day 7 groups. Abbreviations BDL bile duct ligation.

Given the elevated serum copper and decreased FDX1 expression observed in patients, we hypothesized that dysregulated copper homeostasis or cuproptosis might contribute to obstructive cholestatic liver injury. To validate this, we established a BDL rat model to mimic the clinical cholestatic conditions. Seven days after BDL, rats exhibited significantly elevated copper levels in both serum and liver tissue, compared to sham-operated controls (Fig. 2A, B). Compared with both baseline levels at the initiation of modeling and time-matched sham-operated controls, the BDL model group exhibited significant elevations in serum GGT, ALT, and aspartate transaminase (AST) activities, accompanied by a marked rise in TBA concentration (Fig. 2C). Serial sections of liver tissues harvested from rats subjected to BDL for 7 days and time-matched sham-operated controls were stained with H&E, Masson’s trichrome, and dithiooxamide copper-specific stains. Histopathological evaluation revealed distinct peribiliary fibrosis accompanied by parenchymal copper accumulation (green copper salt precipitation) in BDL rats, whereas sham controls maintained normal hepatic architecture with negligible copper deposition (Fig. 2D). Based on the marked elevation of hepatic copper accumulation in BDL models, we further investigated whether copper overload could alter the expression of cuproptosis-related markers in liver tissue. Western blot analysis demonstrated that the expression levels of cuproptosis markers Dihydrolipoamide S-Acetyltransferase (DLAT), Dihydrolipoamide S-Succinyltransferase (DLST), Lipoic Acid Synthetase (LIAS), and FDX1 were downregulated following BDL modeling, which aligns with the characteristic regulatory pattern observed in cells undergoing cuproptosis (Fig. 2E).

To delineate the chronic effects of cholestasis on copper metabolism, we extended the BDL duration to 4 weeks in rats (Supplementary Fig. 2A). Following 4-week modeling, progressive copper accumulation was demonstrated by elevated serum levels (95.1 ± 17.9 μmol/L vs. 13.8 ± 7.2 μmol/L in shams) and elevated hepatic copper content (1.07 ± 0.2 μmol/gprot vs. 0.48 ± 0.07 μmol/gprot in shams; Supplementary Fig. 2B), accompanied by extensive liver injury, bridging fibrosis, parenchymal copper deposition (Supplementary Fig. 2C), and molecular alterations featuring downregulation of FDX1 (Supplementary Fig. 2C–E), with concurrent upregulation of fibrogenic markers collagen 1, vimentin, and α-SMA (Supplementary Fig. 2D).

### High copper intake exacerbates BDL-induced liver injury and fibrogenesis, while TTM ameliorates hepatic pathology in rats

**Fig. 3:**
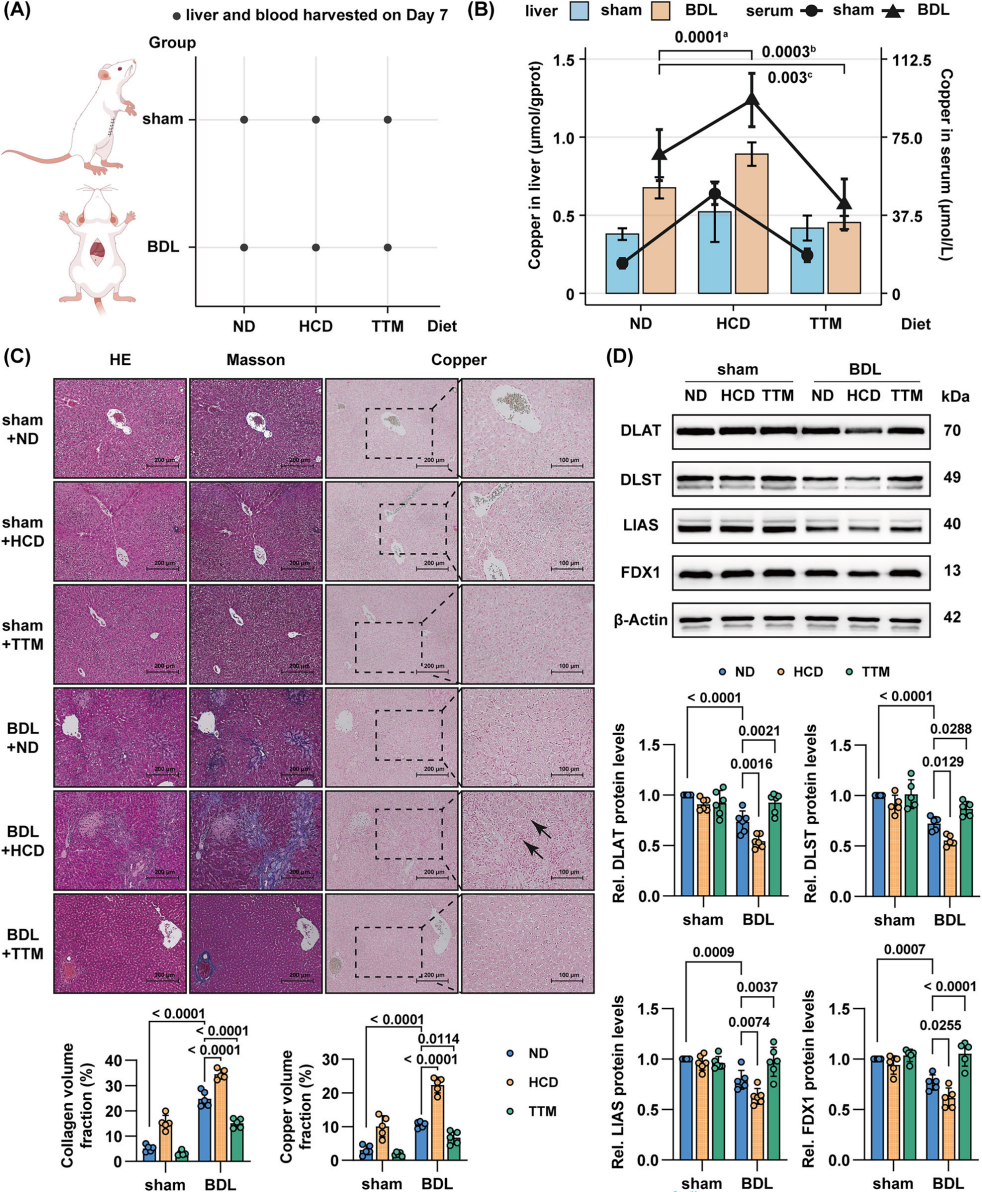
High copper intake exacerbates BDL-induced liver injury and fibrogenesis, while TTM ameliorates hepatic pathology in rats. **A** Schematic diagram of animal experiments. Liver and blood of rats were harvested at 7 days post-operation. **B** Discrepancy in serum and liver copper levels among the groups (*n* = 6). **C** Representative images of serial liver sections stained with H&E, Masson’s trichrome for collagen deposition (blue), and dithiooxamide for copper salt deposits (green granules, *n* = 5). Scale bar = 200 μm. Bottom: fold change in collagen volume fraction and copper volume fraction relative to sham-ND group. **D** Western blot of DLAT, DLST, LIAS and FDX1 in liver tissue of rats (*n* = 5). Bottom: fold change in DLAT, DLST, LIAS and FDX1 expression relative to sham-ND group. Data are mean ± SD. Two-way ANOVA with Šídák’s correction (**C**, **D**). Black arrows denote copper salt deposits. ^a^ Comparing serum values between BDL + HCD and BDL + ND groups. ^b^ Comparing serum values between BDL + TTM and BDL + ND groups. ^c^ Comparing liver values between BDL + TTM and BDL + ND groups. Abbreviations: ND normal diet, NCD high-copper diet, TTM tetrathiomolybdate.

To investigate the pathogenic role of copper in BDL-induced liver injury, we established three experimental subgroups on the BDL rat model (Fig. 3A): normal diet (BDL + ND), high-copper diet (BDL + HCD), and normal diet with copper chelator TTM (BDL + TTM). After 7-day interventions, we observed significant copper accumulation in the BDL + HCD group, with serum copper levels increasing 1.4-fold (92.8 ± 12.8 μmol/L vs. 66.4 ± 12.2 μmol/L in BDL + ND) and hepatic copper content escalating 1.3-fold (0.89 ± 0.08 μmol/gprot vs. 0.68 ± 0.07 μmol/gprot), whereas the BDL + TTM group exhibited 35.8% reduction of serum copper (42.6 ± 12.3 μmol/L) and 32.9% depletion of hepatic copper (0.45 ± 0.04 μmol/gprot) compared to BDL + ND (Fig. 3B). Histopathological evaluation of serial sections demonstrated that HCD exacerbated BDL-induced liver injury, peribiliary fibrosis, and copper deposition, while TTM administration significantly attenuated these pathological changes (Fig. 3C). Notably, compared to the BDL-induced model, where copper predominantly accumulated in the pericellular regions of hepatocytes, a distinct intracellular copper deposition pattern was observed in hepatocytes following the induction of combined BDL and HCD (Fig. 3C). Western blot analysis further confirmed copper-dependent pathway activation, showing HCD-induced downregulation of cuproptosis regulators FDX1, DLAT, DLST, and LIAS, all ameliorated by TTM treatment (Fig. 3D). These results imply a pivotal role of copper overload and cuproptosis in cholestatic liver injury.

### Copper chloride and copper ionophore trigger cuproptosis in rat hepatocytes via copper overload-dependent pathways

**Fig. 4:**
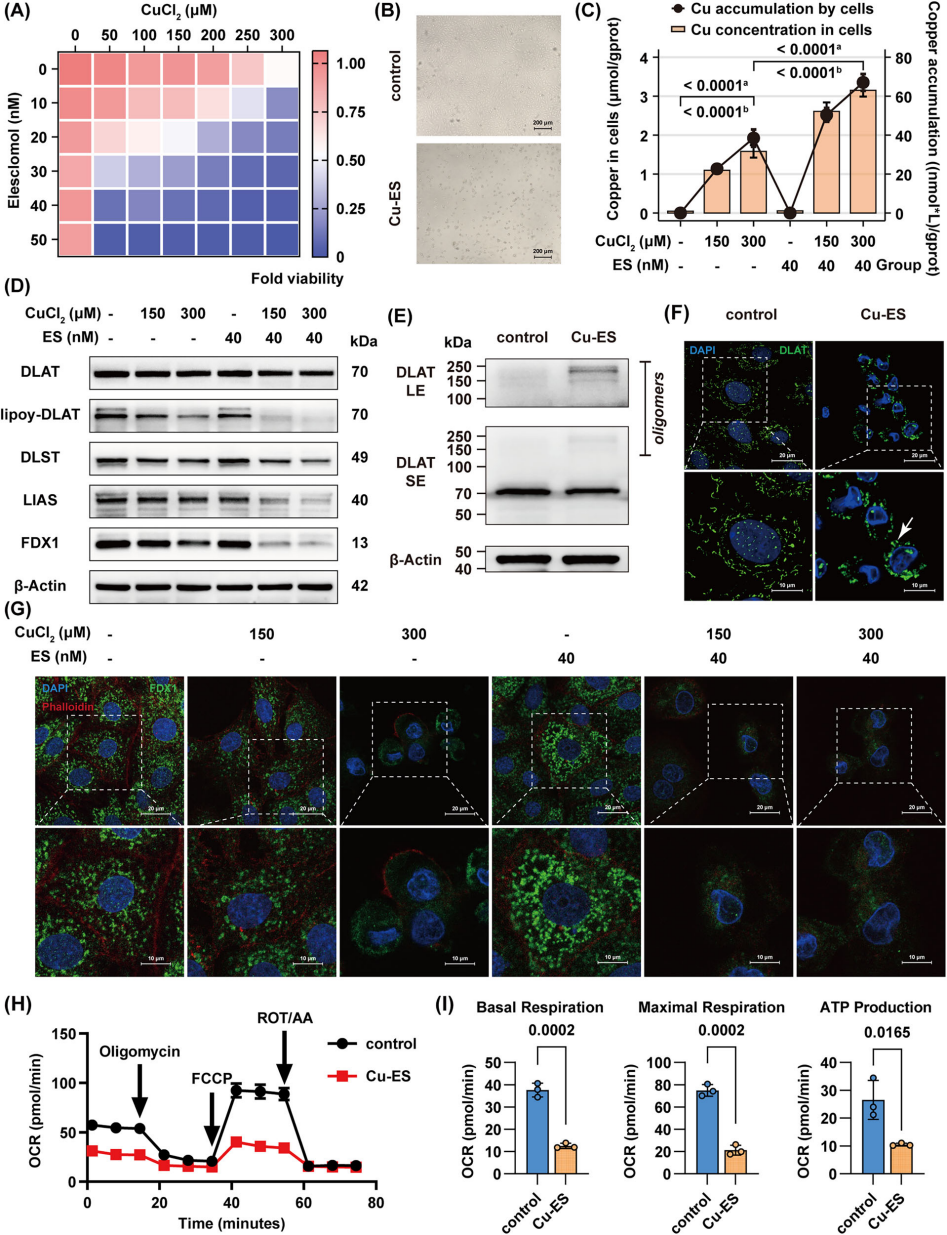
CuCl₂ and copper ionophores induce cuproptosis in rat hepatocytes. **A** Heatmap of CCK-8 assay for cell viability after 24-h CuCl₂ (0 – 300 μM) and ES (0 – 50 nM) treatment, relative to control group (*n* = 3). **B**, **E**, **F**, **H**, **I** BRL 3A cells were treated with CuCl₂ (300 μM) and ES (40 nM) for 24 h. Representative light microscopy images demonstrating cellular morphological changes (**B**, *n* = 3), western blot of monomeric form and oligomerized form of DLAT (**E**, *n* = 3), confocal fluorescence images of DLAT (green) (**F**, *n* = 21–23 cells), and measurement of OCR (**H**, **I** *n* = 3). **C**, **D**, **G** BRL 3A cells were treated with CuCl₂ (0, 150, and 300 μM) and ES (0 and 40 nM) for 24 h. Intracellular copper quantification and copper accumulation quantification (**C**, *n* = 3), western blot of curproptosis-related proteins (**D**, *n* = 3), and merged confocal fluorescence images of FDX1 (green) and Rhodamine phalloidin (red) (**G**, *n* = 10–38 cells). Data are mean ± SD. Two-way ANOVA with Tukey’s correction (**C**) and unpaired Student’s *t* test (**I**). White arrow denotes oligomerized DLAT protein. ^a^ Comparing copper accumulation values between groups. ^b^ Comparing intracellular copper values between groups. Abbreviations: ES elesclomol, LE long exposure, OCR oxygen consumption rate, SE short exposure.

To further determine the role of copper overload and cuproptosis in inducing hepatotoxicity, we established an in vitro model in BRL 3A rat hepatocytes using CuCl₂ combined with the copper ionophore elesclomol (ES) to mimic hepatocytes with copper overload in cholestatic livers. Our findings demonstrated a dose-dependent reduction in cell viability with increasing CuCl₂ concentration gradients (0–300 μM), which was significantly exacerbated by ES concentration gradients (0–50 nM) co-treatment (Fig. 4A). Morphological analysis revealed characteristic changes including cell rounding, cytoplasmic shrinkage, increased nuclear/cytoplasmic ratio, and detachment from culture surfaces (Fig. 4B). Intracellular copper quantification and copper accumulation quantification showed concentration-dependent accumulation that was potentiated by ES (Fig. 4C). Western blot analysis revealed significant downregulation of cuproptosis-related proteins (DLAT, lipoylated DLAT, DLST, LIAS, FDX1; Fig. 4D, Supplementary Fig. 3A). Notably, DLAT oligomerization assays demonstrated decreased monomeric form with concomitant increase in oligomerized form of DLAT (Fig. 4E, Supplementary Fig. 3B). Immunofluorescence confirmed mitochondrial localization of DLAT with reduced expression and increased oligomerization following treatment (Fig. 4F, Supplementary Fig. 3C). Co-staining experiments revealed FDX1 downregulation accompanied by cytoskeletal disintegration (Fig. 4G, Supplementary Fig. 3D). Metabolic profiling showed impaired tricarboxylic acid cycle function, evidenced by reduced expression of DLAT and DLST, coupled with reduced DLAT lipoylation, elevated DLAT oligomerization, and diminished iron-sulfur cluster proteins (Fig. 4D–G). Following copper overload, a significant reduction in basal respiration, maximal respiration, and ATP production was observed in hepatocytes, as measured by the Seahorse oxygen consumption rate (OCR) assay. These results indicate that the aforementioned molecular alterations lead to impaired cellular aerobic respiration and energy metabolism (Fig. 4H, I), which is consistent with the hallmark features of cuproptosis.

**Fig. 5:**
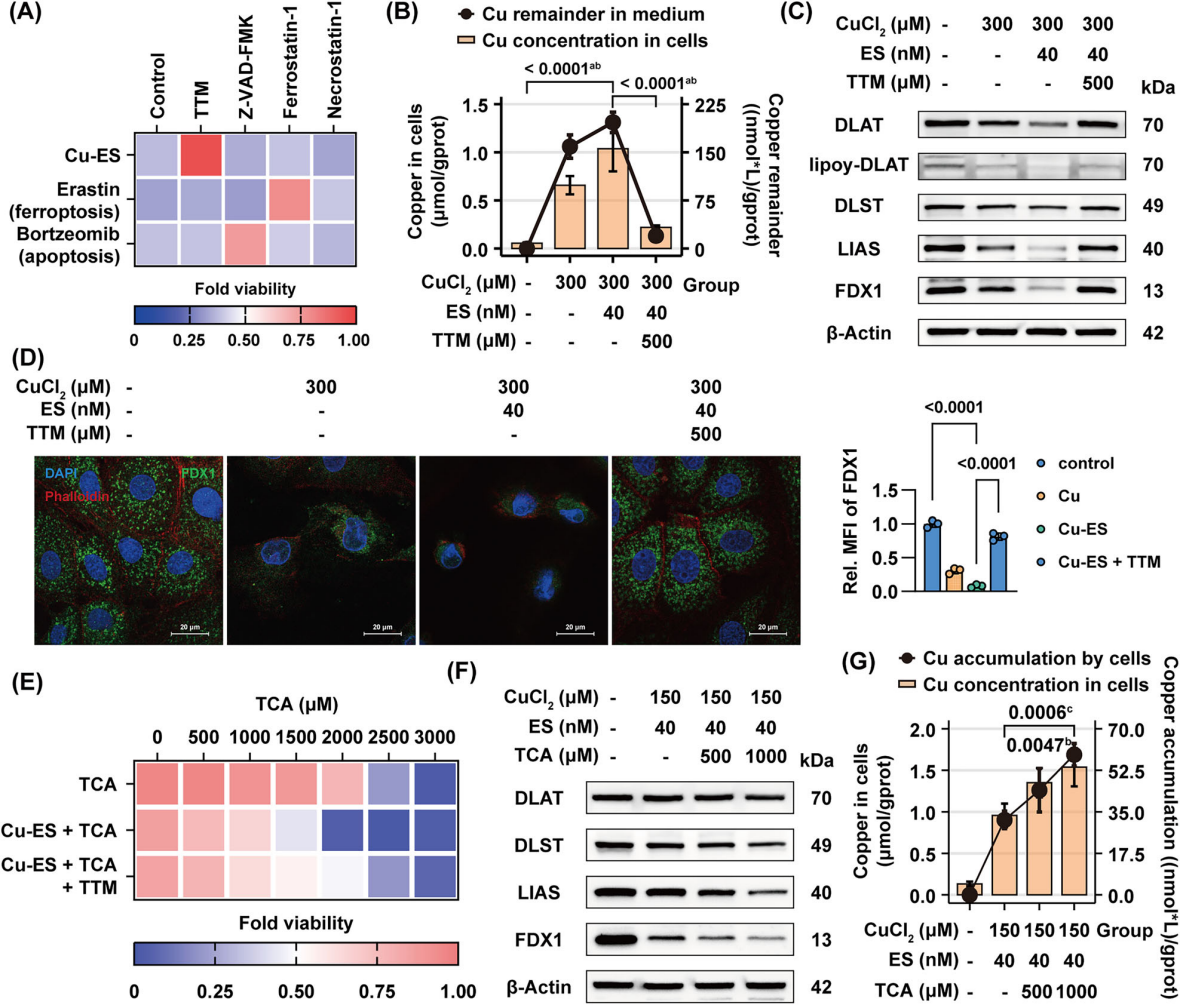
Copper overload induces distinct copper-dependent cell death, and TCA further exacerbates cuproptosis via enhanced copper accumulation. **A** Heatmap of CCK-8 assay for fold cell viability after 24-h CuCl₂ (300 μM)/ES (40 nM), erastin (20 μM), and bortezomib (40 nM) treatment in BRL 3A cells, in combination with respective inhibitors: TTM (500 μM), Z-VAD-FMK (30 μM), ferrostatin-1 (10 μM), and Necrostatin-1 (20 μM), relative to control group (*n* = 3). **B**, **C**, **D** BRL 3A cells were treated with CuCl₂ (300 μM) and ES (40 nM) in combination with TTM (500 μM) for 24 h. Intracellular copper quantification and soluble copper remainder quantification (**B**, *n* = 3), western blot of curproptosis-related proteins (**C**, *n* = 3), and confocal fluorescence images of FDX1 (green) and Rhodamine phalloidin (red) (**D**, *n* = 13–29 cells). Right: fold change in FDX1 MFI relative to the control group. (**E**) Heatmap of CCK-8 assay for cell viability after 24-h CuCl₂ (150 μM)/ES (40 nM)/TTM (500 μM) in combination with TCA (0–3000 μM) treatment, relative to control group (*n* = 3). **F**, **G** BRL 3 A cells were treated with CuCl₂ (150 μM) and ES (40 nM) in combination with TCA (500 or 1000 μM) for 24 h. Western blot of curproptosis-related proteins (**F**, *n* = 3), and intracellular copper quantification and copper accumulation quantification (**G**, *n* = 3). Data are mean ± SD. One-way ANOVA with Bonferroni’s correction (**B**, **G**) and one-way ANOVA with Šídák’s correction (**D**). ^a^ Comparing copper remainder values between groups. ^b^ Comparing intracellular copper values between groups. ^c^ Comparing copper accumulation values between groups. Abbreviations: TCA taurocholic acid, MFI mean fluorescence intensity.

To further investigate the specificity and copper-dependence of copper overload-induced hepatocyte death, we treated BRL 3A cells with multiple inducers and inhibitors of cell death. The results demonstrate that cell death induced by copper chloride (300 μM) and the copper ionophore ES (40 nM) was specifically ameliorated by the copper chelator TTM, with significant viability restoration observed at TTM concentrations more than 300 μM without detectable cytotoxicity (Fig. 5A, Supplementary Fig. 4A). Quantitative analysis revealed that TTM significantly reduced intracellular copper levels and decreased soluble copper content in culture medium (Fig. 5B), indicating that TTM protects hepatocytes by precipitation. Notably, TTM intervention restored protein expression of cuproptosis-related markers, including DLAT, lipoylated DLAT, DLST, LIAS, and FDX1 (Fig. 5C, Supplementary Fig. 4B). Immunofluorescence co-staining further confirmed that TTM attenuated Cu/ES-induced FDX1 downregulation and cytoskeletal disintegration (Fig. 5D, Supplementary Fig. 4C). These findings collectively establish that copper overload triggers a unique form of programmed cell death, mechanistically distinct from ferroptosis and apoptosis, characterized by its unique copper dependency.

### Taurocholic acid exacerbates CuCl₂- and copper ionophore-induced cell death via enhanced copper accumulation

The characteristic pathophysiological distinction of cholestatic liver injury from copper overload-induced damage in other organs lies in the impaired bile acid excretion and subsequent intrahepatic accumulation. To investigate whether bile acids exert additional regulatory effects on copper-induced hepatocyte death under copper overload conditions, we employed TCA, a predominant bile acid species elevated in cholestatic liver [26, 27], in combination with Cu/ES in rat hepatocytes. Our findings demonstrated that TCA significantly exacerbated the Cu/ES-induced decrease in cell viability, an effect that was attenuated by TTM treatment (Fig. 5E). Notably, protein levels of cuproptosis markers showed further downregulation following TCA intervention (Fig. 5F, Supplementary Fig. 4D). Interestingly, quantitative measurements revealed that TCA enhanced both intracellular copper levels and cellular copper accumulation (Fig. 5G). These findings collectively demonstrate that TCA potentiates Cu/ES-induced hepatocyte cuproptosis by promoting cellular copper accumulation, providing novel insights into the pathogenesis of cholestatic liver injury.

### Validation of copper overload-induced and TCA-exacerbated hepatocyte cuproptosis in mouse hepatocytes

To assess phylogenetic conservation of copper overload-induced cuproptosis and TCA-mediated potentiation effects across species, we conducted systematic interventions in mouse AML12 hepatocytes. Dose-dependent viability loss was observed following 24-h combinatorial treatment with CuCl_2_ (0–300 μM) and ES (0–50 nM), with synergistic cytotoxic effects across the concentration spectrum (Supplementary Fig. 5A). Cellular copper accumulation assays revealed increased intracellular copper accumulation under combined treatment (Supplementary Fig. 5B), concomitant with characteristic alterations in cuproptosis biomarker expression profiles that were attenuated by TTM co-treatment (Supplementary Fig. 5C). Subsequent TCA treatment demonstrated exacerbated cuproptosis phenotypes, accompanied by intensified copper accumulation and progressive downregulation of cuproptosis markers (Supplementary Fig. 5D–F). These findings confirm evolutionary conservation of copper overload-mediated cuproptosis and TCA-dependent potentiation effects between mouse and rat hepatocytes.

### Smoothened signaling suppression mediates copper-induced hepatocyte death

**Fig. 6:**
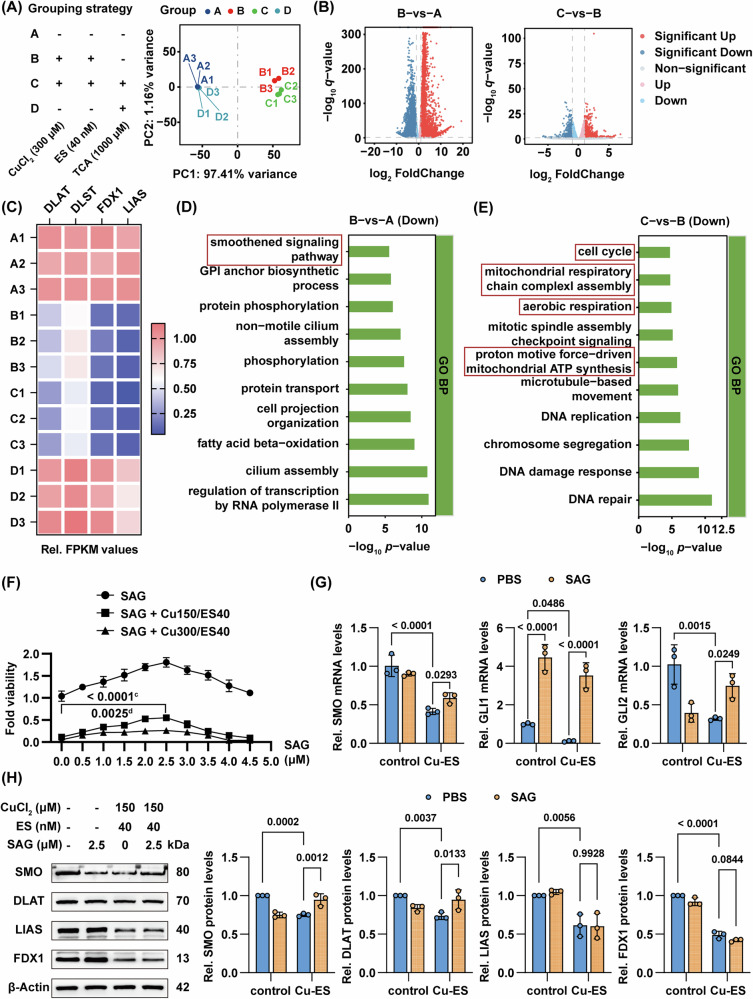
RNA-seq reveals possible molecular mechanisms driving cuproptosis and TCA-mediated exacerbation. **A**, **B**, **C**, **D**, **E** AML12 cells cultured in standard growth medium (Group A), or treated with CuCl₂ (300 μM)/ES (40 nM; Group B), or treated with CuCl₂ (300 μM)/ES (40 nM)/TCA (1000 μM; Group C), or treated with TCA (1000 μM; Group D) for 24 h. Grouping strategy and PCA of RNA-sequencing data among the four groups (**A**), volcano plot of DEGs between groups (**B**), heatmap of standardized FPKM values of cuproptosis-related genes, normalized to group A (**C**), and GO pathway analysis of DEGs (|log2 FC| > 1 and *q* value < 0.05), showing the top 10 enriched biological processes (**D**, **E**). **F**, **G** BRL 3A cells were treated with SAG, or treated with SAG/CuCl₂ (150 μM)/ES (40 nM), or treated with SAG/CuCl₂ (300 μM)/ES (40 nM) in SAG concentration gradients (0–4.5 μM) for 24 h. Line plots of CCK-8 assay for fold cell viability, relative to control group (**F**; *n* = 3), and RT-qPCR of SMO, GLI1 and GLI2, normalized to the control group (**G**; *n* = 3). **H** Western blot of DLAT, LIAS, FDX1 and SMO after BRL 3A cells were treated with CuCl₂ (150 μM) and ES (40 nM) in combination with SAG (2.5 μM) for 24 h. Right: fold change in protein expression relative to control group. Data are mean ± SD. One-way ANOVA with Šídák’s correction (**F**) and two-way ANOVA with Šídák’s correction **G**, **H** Abbreviations: PCA principal component analysis, DEGs differentially expressed genes, FPKM fragments per kilobase million, SAG smoothened agonist.

Given that cuproptosis is primarily regulated through post-translational mechanisms, we further investigated the transcriptional alterations underlying copper overload-induced cuproptosis and TCA-mediated exacerbation of this process. Considering the comprehensive genomic annotation and well-established genetic tools available for mouse models, we performed RNA sequencing on treated AML12 mouse hepatocytes. Principal component analysis (PCA) revealed substantial transcriptomic reprogramming in Cu/ES-treated cells compared to controls (group B vs. A, Fig. 6A), with additional divergence upon TCA co-treatment (group C vs. B, Fig. 6A). However, TCA monotherapy showed minimal transcriptional alterations relative to controls (group D vs. A, Fig. 6A). Differential expression analysis (*q* < 0.05, |log2FC| > 1) identified upregulated and downregulated genes between groups (Fig. 6B). Quantitative analysis of standardized fragments per kilobase million (FPKM) values revealed that key cuproptosis-related genes (DLAT, DLST, FDX1, and LIAS) exhibited downregulation following induction with Cu/ES (Fig. 6C). Notably, this suppressive effect was potentiated upon TCA intervention, validating the above-mentioned results in RNA-seq data (Fig. 6C). Given the characteristic downregulation of cuproptosis markers at the protein level, Gene Ontology biological process (GO-BP) analysis of downregulated genes in Cu/ES-treated cells revealed significant enrichment in the smoothened signaling pathway (GO:0007224), implying a novel regulatory axis in cuproptosis (Fig. 6D). GSEA also revealed statistically significant enrichment of the smoothened signaling pathway (Normalized ENS = –1.84, FDR = 0.015), with the characteristic negative enrichment score peak (minimum ENS = –0.537) demonstrating coordinated transcriptional suppression of core components in this signaling cascade (Supplementary Fig. 5G). Notably, TCA co-treatment was specifically enriched in mitochondrial-related processes, including mitochondrial respiratory chain complex I assembly (GO:0032981), aerobic respiration (GO:0009060), and proton motive force-driven ATP synthesis (GO:0042776) among downregulated genes (Fig. 6E), indicating amplified mitochondrial dysfunction that aligns with and intensifies characteristic cuproptosis pathology.

To delineate the regulatory role of smoothened (SMO) signaling pathway in cuproptosis, we conducted comparative interventions in rat BRL 3A and mouse AML12 hepatocytes under Cu-ES induction with/without SAG. SAG co-treatment significantly ameliorated the cytotoxic effects (Fig. 6F, Supplementary Fig. 5H). RT-qPCR and western blot confirmed copper-induced downregulation and SAG-mediated restoration of SMO, accompanied by rescue of GLI1 and GLI2, the downstream regulators of SMO (Fig. 6G, H, Supplementary Fig. 5I). Meanwhile, the expression of DLAT was restored by SAG while FDX1 and LIAS remained suppressed (Fig. 6H). These results demonstrate the role of smoothened signaling pathway in cuproptosis, and the therapeutic effects of SMO activation through DLAT-dependent mechanisms, which are independent of FDX1 in the cuproptosis cascade.

### FDX1 plays a complex regulatory role in hepatocyte cuproptosis

**Fig. 7:**
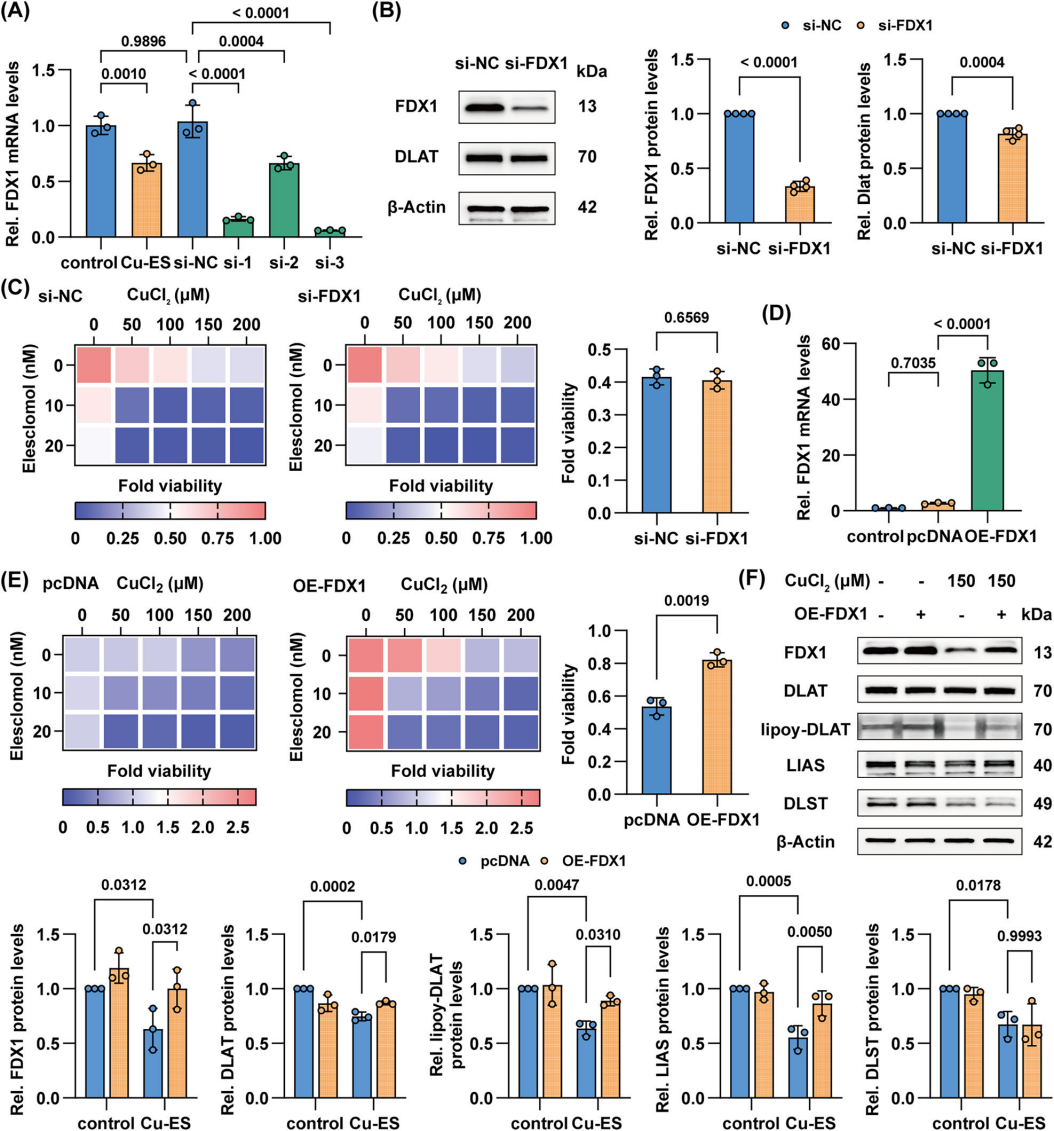
FDX1 plays a complex regulatory role in hepatocyte cuproptosis. **A** RT-qPCR of FDX1 after BRL 3A cells treated with CuCl₂ (300 μM)/ES (40 nM) or siRNA negative control (si-NC; 50 nM) or three distinct siRNA constructs against FDX1 (si-FDX1; 50 nM) for 24 h, normalized to the control group (*n* = 3). **B** Western blot of FDX1 after BRL 3 A cells were treated with si-NC (50 nM) or si-3 (50 nM) for 24 h. Right: fold change in protein expression relative to control group. **C** Heatmap of CCK-8 assay for cell viability after 24-h CuCl₂ (0–200 μM)/ES (0–20 nM) in combination with si-NC (50 nM) or si-3 (50 nM) for 24 h, relative to si-NC control group. Right: comparative analysis of cell viability of Cu150/ES0 subgroups. **D** RT-qPCR of FDX1 after BRL 3A cells were treated with pcDNA 3.1 (+) (pcDNA; 6 μg/well in the 6-well plate) or FDX1-expressing plasmid (OE-FDX1; 6 μg/well in the 6-well plate) for 24 h, normalized to the control group (*n* = 3). **E** Heatmap of CCK-8 assay for cell viability after 24-h CuCl₂ (0–200 μM)/ES (0–20 nM) in combination with pcDNA (6 μg/well in the 6-well plate) or OE-FDX1 (6 μg/well in the 6-well plate) for 24 h, relative to pcDNA control group. Right: comparative analysis in cell viability of Cu150/ES0 subgroups. **F** Western blot of FDX1, DLAT, lipoylated-DLAT, LIAS, and DLST after BRL 3A cells were treated with CuCl₂ (150 μM) in combination with pcDNA (6 μg/well in the 6-well plate) or OE-FDX1 (6 μg/well in the 6-well plate) for 24 h. Bottom: fold change in protein expression relative to control-pcDNA group. Data are mean ± SD. One-way ANOVA with Šídák’s correction (**A**, **D**), unpaired Student’s *t* test (**B**, **C**, **E**), and two-way ANOVA with Šídák’s correction (**F**).

**Fig. 8:**
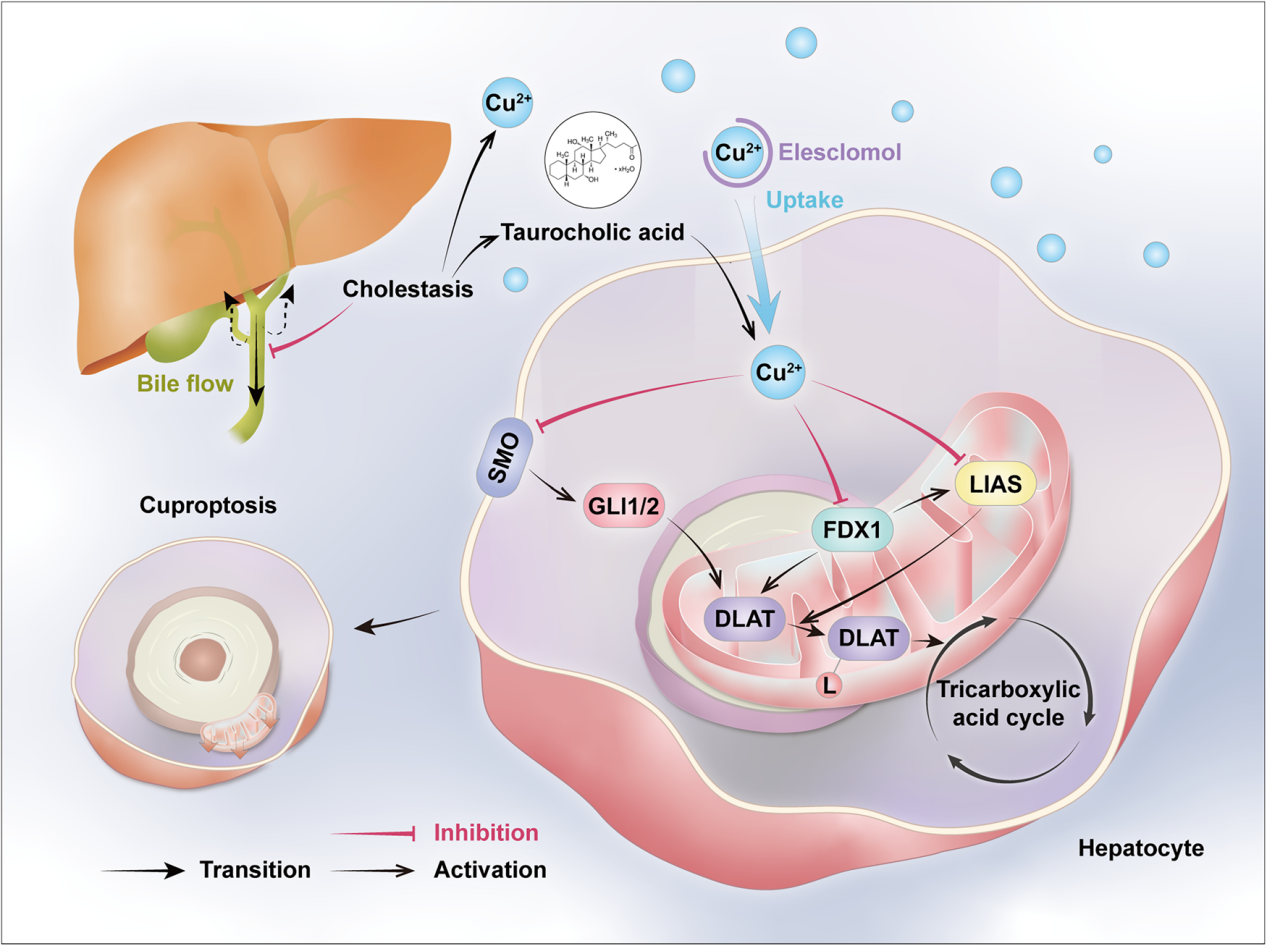
A schematic diagram illustrates a novel cuproptosis-mediated pathogenic mechanism driving cholestatic liver injury. Initially, cholestasis raises copper levels within the liver and causes copper overload of hepatocytes. Meanwhile, concurrent accumulated TCA further enhances copper accumulation. The accumulated copper subsequently triggers dual regulatory effects: suppression of FDX1 expression and inhibition of the smoothened signaling pathway. These molecular alterations culminate in reduced LIAS expression and diminished DLAT lipoylation. Ultimately, malfunction of tricarboxylic acid cycle leads to programmed cell death of hepatocytes.

To investigate the precise regulatory role of cuproptosis-related key gene *FDX1* in hepatocytes, we performed both loss-of-function (using three distinct siRNAs targeting FDX1 mRNA) and gain-of-function (via FDX1-expressing plasmid) experiments. In the knockdown studies, RT-qPCR analysis revealed significant downregulation of FDX1 mRNA levels across all siRNA constructs, with si-3 demonstrating the highest knockdown efficiency at equivalent concentrations, thus being selected for subsequent experiments (Fig. 7A). Western blot confirmed reduced FDX1 protein expression following si-FDX1 treatment, concomitant with decreased DLAT protein levels, suggesting a potential regulatory effect of FDX1 on DLAT (Fig. 7B). Notably, CCK-8 assays under the exact experimental conditions showed that FDX1 knockdown did not enhance cellular resistance to cuproptosis (Fig. 7C). In complementary gain-of-function studies, FDX1 mRNA levels were significantly elevated post-transfection with the FDX1-expressing plasmid (Fig. 7D). Intriguingly, FDX1 overexpression significantly enhanced hepatocyte resistance to cuproptosis (Fig. 7E). Mechanistically, western blot analysis under copper overload conditions demonstrated upregulated protein expression of DLAT, LIAS, and lipoylated DLAT in FDX1-overexpressing cells, providing a potential molecular basis for this phenotypic observation (Fig. 7F). The integrated mechanism network elucidated in the current investigation was schematically illustrated in Fig. 8.

## Discussion

Copper is predominantly excreted via hepatic biliary elimination under physiological conditions, whereas cholestatic conditions induce hepatic copper retention, leading to copper overload in the liver and concomitant elevation of serum copper levels. Whether such copper overload triggers hepatocyte cuproptosis and subsequently contributes to liver injury pathogenesis remains to be fully elucidated. Here, we demonstrated that copper overload acts as a cholestasis-associated event, triggering hepatocyte cuproptosis. Concurrent hepatic TCA accumulation synergistically exacerbated copper-induced hepatotoxicity through facilitating cellular copper accumulation. Mechanistic investigations identified FDX1 as a pivotal upstream regulator of cuproptosis, while the suppression of the smoothened signaling pathway was implicated in cuproptosis pathogenesis. Therapeutically, copper chelators effectively treated cholestasis-induced copper overload hepatotoxicity both in vivo and in vitro, whereas pharmacological activation of smoothened signaling using SAG effectively reversed copper-induced cuproptosis alterations, providing a therapeutic avenue for cholestatic liver injury.

Despite established evidence that copper overload in cholestatic livers [12] and chronic copper deposition serve as a definitive pathogenic factor driving hepatic fibrosis in Wilson’s disease [10], the mechanistic role of hepatic copper overload in acquired cholestatic liver injury remains elusive. Our study reveals the direct involvement of copper-induced cuproptosis in cholestatic hepatocyte death. Clinical biochemistry analysis of human hospital attendees and rat BDL models demonstrated concomitant elevation of serum and/or hepatic copper levels with cholestatic injury progression. Notably, BDL-operated rats exhibited significant alterations in cuproptosis-related biomarker expression. Experimental modulation through high-copper diet or TTM chelation therapy, respectively, exacerbated or ameliorated BDL-induced hepatotoxicity. In vitro validation showed that CuCl_2_ and copper ionophore specifically induced intracellular copper accumulation and triggered a unique cell death pattern distinguishable from ferroptosis, apoptosis, and necroptosis in rodent hepatocytes—a process effectively inhibited by TTM co-treatment. These findings collectively establish copper-mediated hepatocyte cuproptosis as a critical pathogenic mechanism in cholestatic liver injury.

The proper lipoylation and functional integrity of key proteins involved in cellular energy metabolism are essential for maintaining mitochondrial homeostasis and ensuring efficient energy production processes [32]. *DLAT* encodes dihydrolipoyl transacetylase, the E2 catalytic enzyme of eukaryotic pyruvate dehydrogenase complex (PDHc). The lipoylated E2 subunit of PDHc catalyzes the transfer of the acetyl group from pyruvate to coenzyme A (CoA), resulting in the formation of acetyl-CoA, which subsequently enters the tricarboxylic acid cycle [33]. *DLST* encodes the E2 subunit of the α-ketoglutarate dehydrogenase complex (α-KGDH) in its lipoylated form, acts as the catalytic core responsible for the decarboxylation of 2-oxoglutarate to succinyl-CoA and CO₂ within the tricarboxylic acid cycle [34]. Copper overload downregulates total protein expression and impairs lipoylation of DLAT, while concurrently promoting DLAT oligomerization. These pathological alterations collectively disrupt cellular energy metabolism by compromising mitochondrial enzymatic complexes, triggering depletion of iron-sulfur cluster proteins, and ultimately culminating in programmed cell death.

FDX1, a reductase known to reduce Cu^2+^ to Cu^+^, serves as the key regulator of coproptosis [19], the role of which was primarily discovered by Genome-wide CRISPR/Cas9 screening [35]. As reported in multiple studies, FDX1 was down-regulated after copper intervention [36, 37], while the exact regulatory effect on copper-induced cell death in cholestatic liver disease remains to be explored. We performed loss and gain of function of FDX1 under normal and cuproptosis conditions. Interestingly, after remarkable decreased and increased expression of FDX1 in mRNA and protein, siRNA-mediated FDX1 knockdown exhibited no marked effect on cell viability in hepatocytes, while plasmid-driven FDX1 overexpression rescued the viability of cuproptic hepatocytes. Consistent with previous reported studies that FDX1 promotes lipoylation of DLAT [38, 39], we found that after overexpression of FDX1, the monomeric and lipoylated form of DLAT were upregulated, which may outweigh the toxic effects of FDX1 overexpression and serves as a possible explanation for the exact phenomenon in hepatocytes. The exact mechanisms and underlying regulatory networks require further investigation.

The distinct feature of cholestatic copper overload lies in the coexistence of copper overload and accumulated bile components within the hepatic microenvironment. Consistent with previous studies that excess bile acids, including TCA, in chronic cholestasis mouse models impaired adipose mitochondrial function and caused defective thermogenesis, which implies the correlation between bile acid overload and mitochondrial dysfunction [40]. In this study, we investigated the role of TCA, the predominant and markedly elevated bile acid in cholestatic livers [26, 27], in hepatocyte cuproptosis. Notably, under copper overload conditions, TCA significantly exacerbated cell death at substantially lower concentrations and induced characteristic alterations in cuproptosis biomarkers, primarily through enhanced cellular copper accumulation. Furthermore, enrichment analysis of RNA-seq data revealed that downregulated mRNAs in TCA-treated hepatocytes were predominantly associated with aerobic respiration and energy metabolism-associated pathways, indicating amplified cuproptosis features post-TCA exposure. These findings collectively suggest that TCA plays a pivotal role in aggravating copper overload-induced hepatocyte cuproptosis during cholestasis. This insight warrants further mechanistic investigations to elucidate the precise molecular mechanisms involved.

Although copper-induced cytotoxicity primarily occurs through post-translational gene regulatory mechanisms [19], we investigated transcriptional alterations in cuproptotic hepatocytes. Enrichment analysis revealed a critical involvement of the smoothened signaling pathway in copper-induced cell death. This pathway is essential in embryonic development [41], particularly neurogenesis [42], and has demonstrated neuroprotective effects in glucocorticoid-induced neonatal cerebellar injury [43]. Notably, SAG was reported to mitigate mitochondrial dysfunction in frataxin-deficient astrocytes, manifested by restored maximal respiratory capacity, basal mitochondrial respiration, and spare respiratory capacity [44], suggesting smoothened activation could reverse compromised mitochondrial respiration. Our experimental validation confirmed that SAG treatment effectively reversed copper-induced DLAT downregulation, potentially restoring mitochondrial functional integrity and significantly enhancing cell viability, thereby identifying smoothened pathway modulation as a promising therapeutic strategy against hepatic cuproptosis.

In summary, our findings reveal the pivotal role of copper overload-induced cuproptosis in cholestatic liver injury. This process is exacerbated by spatiotemporally synchronized accumulation of taurocholic acid, which potentiates hepatocyte copper accumulation. Mechanistically, FDX1 emerges as the critical mediator of cuproptosis in hepatocytes, where FDX1 upregulation confers protection against copper overload-induced cell death. Transcriptomic analysis demonstrates that this copper-mediated cell death process requires Smoothened signaling pathway regulation. Therapeutically, copper chelator TTM demonstrates significant efficacy in attenuating copper overload-induced cytotoxicity both in vitro and in vivo. Notably, SAG administration restores DLAT protein expression and improves cellular viability. These results establish a direct link between FDX1-mediated taurocholic acid-exacerbated cuproptosis and cholestasis-induced hepatotoxicity, providing both theoretical and experimental foundations for developing novel therapeutic strategies against cholestatic liver injury.

## Materials and methods

### Patients

The retrospective clinical study was approved by the Ethical Committee of the Sixth Affiliated Hospital of Harbin Medical University (Approval No. LC2024-114). The requirement for informed consent was waived by the Ethics Committee because the study used fully de-identified extensive laboratory data and posed minimal risk to participants. All data were anonymized by removing personal identifiers prior to analysis, in compliance with the Declaration of Helsinki and Istanbul [45, 46].

Clinical test result data were retrospectively collected from August 2022 through March 2025. For analysis, complete results of blood test items performed on the same day for the same patients were included. All the de-identified blood test records were listed in Supplementary Table 1.

### Single-cell RNA-sequencing of BA datasets analysis

Single-cell RNA sequencing (scRNA-seq) datasets derived from liver tissues of BA patients and controls were retrieved from the Gene Expression Omnibus (GEO) database (accession: GSE176189), incorporating three BA samples (GSM5359592, GSM5359593, GSM5359594) and three control samples (GSM5359607, GSM5359608, GSM5359609) for integrative analysis. Downstream processing was performed using the Seurat R package (version 5.2.1) following standardized workflows, with group-wise average gene expression levels calculated via the AverageExpression function within the Seurat framework.

### Reagents

Copper (II) chloride (CuCl_2_) dihydrate (Cat# C3279) and ammonium tetrathiomolybdate (TTM, Cat# 323446) were purchased from Sigma-Aldrich (St. Louis, MO, USA). Elesclomol (ES, Cat# HY-12040), Erastin (Cat# HY-15763), and Ferrostatin-1 (Cat# HY-100579) were from MedChemExpress (Shanghai, China). TCA sodium salt hydrate (Cat# ST1690), Bortezomib (Cat# SC0263), Z-VAD-FMK (Cat# C1202), Necrostatin-1 (Cat# SC4359), and Smoothened agonist (SAG, Cat# SF6836) were from Beyotime (Shanghai, China).

FDX1 siRNA and its transfection reagent were synthesized by RiboBio (Guangzhou, China), with the siRNA-targeting sequences provided in Supplementary Table 2. The FDX1-expressing plasmid, constructed in the pcDNA3.1(+) vector, along with its transfection reagent, was obtained from GenePharma (Suzhou, China). Transfection procedures for both siRNA and plasmids were performed following the manufacturer’s protocols.

### Animals

All experimental procedures were performed following the Animal Ethics Committee guidelines of the Sixth Affiliated Hospital of Harbin Medical University (Approval No. LC2024-114). Specific pathogen-free male SD rats (4 –5 weeks old) were purchased from the Animal Center of Harbin Medical University. All the rats were housed in a temperature-controlled environment on a 12-h light-dark cycle with free access to water and diet. SD rats were randomized to each group using a computer-generated random number sequence.

### Animal procedure

The bile duct ligation (BDL) procedure was performed as described [47]. Briefly, SD rats were anesthetized with intraperitoneal pentobarbital (4 mg/kg), and the abdomen was sterilized and incised to expose the bile duct. The common bile duct was then ligated twice with 4–0 silk in the upper and lower regions. Rats in the sham group underwent the same procedure without BDL. After a maximum of 4 weeks, the rats were euthanized and their livers and serum were harvested.

The high-copper diet for rats was custom-formulated by Keao Xieli (Tianjin) Feed Co., Ltd. with the copper concentration set at 10 times higher (100–200 mg/kg) than that of a standard rat diet (10–20 mg/kg) [48], while all other components were maintained unchanged. In the high-copper diet group, following BDL modeling, rats were fully transitioned to the high-copper diet. TTM was administered via gavage at a dose of 10 mg/kg [49], dissolved in deionized water. It was applied daily to the rats in the TTM group following the BDL procedure. The rats had free access to food and water throughout the experiment.

### Copper quantification in liver tissue

For quantification of copper content in liver tissues, a kit (Cat# E-BC-K300-M, Elabscience) was used following the manufacturer’s recommended protocols. Briefly, following thorough homogenization of liver tissues in double-distilled water, the lysates were centrifuged at 12,000 × *g* for 10 min. The resulting supernatant was carefully collected and reacted with freshly prepared chromogenic working solution at 37 °C for 5 min. Absorbance measurements at 580 nm were subsequently conducted for absolute copper quantification. An aliquot of the supernatant was subjected to protein concentration determination using the BCA assay. The absolute copper concentration was normalized to the corresponding protein concentration, which served as the quantitative measure of copper content in liver tissue.

### Histopathology and immunofluorescent staining

Liver tissues harvested from SD rats were fixed in 4% paraformaldehyde for 48 h, paraffin-embedded, and then sectioned at 4 μm thickness.

For H&E staining, sections were dewaxed, hydrated, stained with Mayer’s hematoxylin and eosin Y, then dehydrated through graded ethanol before neutral balsam mounting. For Masson’s trichrome staining, after Weigert’s iron hematoxylin nuclear staining, sections were sequentially treated with Biebrich scarlet-acid fuchsin, phosphomolybdic acid, and aniline blue, followed by 1% acetic acid differentiation. For copper-salt staining, the dithiooxamide method (copper salt stain kit, Cat# G3040, Solarbio) was performed according to the manufacturer’s instructions. Briefly, deparaffinized sections were incubated with dithiooxamide staining solution, counterstained with nuclear fast red solution, and then conventionally dehydrated and transparentized. Images were analyzed using ImageJ (version 1.54p) to quantify fibrosis or copper-positive areas.

For immunofluorescent staining, paraffin-embedded rat liver sections underwent antigen retrieval via citrate buffer. After blocking with 5% BSA/PBS, sections were incubated with rabbit anti-FDX1 antibody, followed by FITC-conjugated goat anti-rabbit IgG. Nuclear counterstaining utilized DAPI (Cat# C1006, Beyotime). Slides were mounted with anti-fade medium (Cat# P0126, Beyotime) and imaged using a Zeiss fluorescence microscope. Images were analyzed using ImageJ to quantify MFI. Specific antibodies are listed in Supplementary Table 3.

### Cells

The rat hepatocytes BRL 3A and mouse hepatocytes AML12 were purchased from the National Collection of Authenticated Cell Cultures (Shanghai, China). The cells were authenticated by short tandem repeat (STR) profiling within the past 6 months and confirmed to be free of mycoplasma contamination prior to experimentation. BRL 3A cells were cultured in DMEM (Gibco, USA) supplemented with 10% FBS (ExCell Bio, China) and 1% penicillin-streptomycin (Beyotime). AML12 cells were cultured in DMEM/F12 (1:1) (Gibco) supplemented with 10% FBS (ExCell Bio), 1% ITS (insulin-transferrin-selenium) liquid media supplement (I3146, Sigma), and 1% penicillin-streptomycin (Beyotime). All the cells were cultured in a 5% CO_2_ atmosphere at 37 °C.

### Cell procedure

For drug addition, where indicated, a specific dose of CuCl_2_ dihydrate, ES, ammonium TTM, TCA, SAG, or any other reagents was added to the culture medium 30 min prior to treatment to achieve the target final concentration.

### Cellular copper quantification

For quantification of cellular copper content, a kit (Cat# E-BC-K775-M, Elabscience) was used following the manufacturer’s recommended protocols. Briefly, cells were lysed with lysis buffer on ice for 10 min and then centrifuged at 12,000 × *g* for 10 min. The supernatant was mixed with freshly prepared chromogenic working solution and incubated at 37 °C for 5 min. The absorbance was subsequently measured at a wavelength of 580 nm for the calculation of absolute copper concentration. Simultaneously, an aliquot of the supernatant was reserved for protein concentration determination using the BCA assay. The absolute copper concentration was normalized to the protein concentration to determine the intracellular copper relative level, which served as the quantitative measure of intracellular copper content.

For quantification of cellular net copper accumulation, absolute copper concentration in the conditional medium was obtained with the same method used in cell lysis. Absolute copper accumulation was calculated by multiplying the volume of culture medium by the differential value between the initial modeling concentration specified in the medium and the absolute copper concentration measured in the conditioned medium post-modeling. The relative copper accumulation, serving as the cellular copper accumulation, was determined through normalization of the absolute copper accumulation to the cellular protein concentration. In experimental systems with TTM intervention, where TTM chelates copper ions into insoluble precipitates, the copper remainder was alternatively determined by normalizing the absolute copper concentration measured in the conditioned medium post-modeling to the total cellular protein concentration. All negative values approaching zero derived from computational calculations were algorithmically corrected to zero throughout the data processing workflow.

### Bulk RNA-sequencing analysis

AML12 cells were harvested after the indicated treatment (*n* = 3) and total RNA was extracted. Library preparation, quality control, and sequencing were performed by OE Biotech (Shanghai, China). RNA sequencing was performed via the Illumina Novaseq 6000 platform. Differential expression analysis was performed with R package DEseq2 (version 1.22.2) with a threshold of *q* value < 0.05 and |log2FC| > 1. Gene ontology (GO) enrichment analysis was performed by R package clusterProfiler (version 4.8.1). Gene Set Enrichment Analysis (GSEA) was performed using GSEA software (version 4.4.0).

### Statistics

Data were analyzed by R (version 4.4.1) or GraphPad Prism 9 and were presented as the mean ± SD. Normality of data was tested using the Shapiro‒Wilk test. The homogeneity of variances for the data was assessed using the Brown-Forsythe test. For two-group comparisons, normally distributed datasets were subjected to the unpaired Student’s *t* test; non-normally distributed datasets were subjected to the Mann‒Whitney test. For multiple comparisons, normally distributed datasets were subjected to one-way or two-way ANOVA, followed by the Tukey, Bonferroni, or Šídák test for post hoc comparisons when appropriate, and non-normally distributed datasets were subjected to the Kruskal‒Wallis test followed by Dunn’s multiple comparisons test. All data were analyzed using two-tailed tests, and a *p*-value of less than 0.05 was considered statistically significant.

## Supplementary information

Supplementary information

Supplementary Table 1

## Data Availability

The RNA high-throughput sequencing data have been uploaded to the GEO database (GSE300382). Data supporting the present study are available from the corresponding author upon reasonable request. Original images of unprocessed western blot are available at Supplementary information.
