# Supplementary material for: FDX1-mediated cuproptosis promotes cholestatic liver injury exacerbated by taurocholic acid-enhanced copper accumulation
Source: Cell Death Discov. 2026 Jan 9;12:12. doi: 10.1038/s41420-025-02861-7 (PMC12789603; doi:10.1038/s41420-025-02861-7)

***Supplementary information***

**FDX1-mediated cuproptosis promotes cholestatic liver injury exacerbated by taurocholic acid-enhanced copper accumulation**

Yujun Guo^1^, Min Yang^1^, Shengbo Sun^1^, Zhaohua Zhong^2^, Wenjun Lu^1,3^, Ze’nan Zhang^1^, Meili Fan^1^, Aodan Zhang^4^, Tingting Zhang^5^, Yang Wu^6^, Zhou Li^1^, Zuwei Liu^7^, Qijun Sun^1^, Zhaozhu Li^1^, Qingbo Cui^1^*

^1^Department of Pediatric Surgery, The Sixth Affiliated Hospital of Harbin Medical University, Harbin 150000, China

^2^Department of Microbiology, Harbin Medical University, Harbin 150000, China

^3^Laboratory of Systems Immunology, Institute of Basic Medical Sciences, Westlake Institute for Advanced Study, Hangzhou 310024, China.

^4^Department of Pediatric Surgery, Women and Children's Hospital, School of Medicine, Xiamen University, Xiamen 361003, China.

^5^Psychology and Health Management Center, Harbin Medical University, Harbin 150000, China.

^6^Department of Pediatric Surgery, The Second Affiliated Hospital of Harbin Medical University, Harbin 150000, China

^7^Department of Hematology, The Second Affiliated Hospital of Harbin Medical University, Harbin 150000, China

*Lead contact

**Summary**

Supplementary methods, figures and figure legends, tables, and original western blots of the study.

**Supplementary Methods**

**Cell viability**

Cell viability was assessed by incubating cells with CCK-8 reagent (Cat# E-CK-A362, Elabscience) at 37°C for 1 h, followed by absorbance measurement at 450 nm by a microplate reader.

**Immunocytochemistry**

BRL 3A hepatocytes were fixed with 4% paraformaldehyde (15 min), permeabilized with 0.1% Triton X-100 (10 min), and blocked with 5% goat serum (1 h) before sequential staining with rabbit anti-FDX1 (1:200, 4°C overnight), FITC-conjugated secondary antibody (1:500, 1 h), and either Rhodamine Phalloidin (1:50, 1 h mixed with the secondary antibody; Cat# RM02835, Abclonal) for cytoskeletal visualization or MitoTracker Red CMXRos (100 nM, 45 min pre-fixation; Cat# C1049B, Beyotime) for mitochondrial labeling, followed by DAPI counterstaining (5 μg/mL, 10 min; Cat# C1006, Beyotime). Coverslips were mounted with antifade medium (Cat# P0126, Beyotime) and imaged using a Zeiss fluorescence microscope. Images were analyzed using ImageJ (version 1.54p) to quantify mean fluorescence intensity (MFI). Specific antibodies are listed in Supplementary Table 3.

**Blood tests**

Serum was collected by centrifugation at 3,000 rpm for 15 min at 4℃. Activities of ALT, AST, and GGT, and concentration of copper and TBA were quantitatively analyzed using assay kits (Cat# E-BC-K235-M for ALT, Cat# E-BC-K236-M for AST, Cat# E-BC-K126-M for GGT, and Cat# E-BC-K181-M for TBA, all from Elabscience) following the manufacturer's recommended protocols.

**Seahorse XF cell energy metabolism assay**

Measurement of cellular oxygen consumption rate (OCR) was performed according to previously described methods.^1^ Briefly, BRL 3A cells were seeded into XFe96 cell culture microplates (Agilent). After 24 hours of copper overload induction, the culture medium was discarded and replaced with Seahorse XF Base Medium (Agilent) supplemented with 10 mM glucose, 2 mM glutamine, and 1 mM pyruvate, followed by a 1-hour pre-incubation. Subsequently, oligomycin, p-trifluoromethoxy carbonyl cyanide phenylhydrazone, and rotenone/antimycin A were sequentially injected to assess OCR. Data were analyzed using the Seahorse XFe96 Wave Software.

**Western blot and real-time quantitative PCR**

Total protein was extracted from freshly harvested liver tissues and cells and processed as described.^2^ Specific antibodies are listed in Supplementary Table 3. Total RNA was extracted from freshly harvested liver tissues and cells and was processed as described.^2^ Specific primers are listed in Supplementary Table 4.

**Sample size calculation**

The sample sizes for *in vitro* (*n* = 3) and *in vivo* (*n* = 5 – 6) experiments were selected based on established conventions in the field. A post-hoc power analysis confirmed that the achieved power for the key findings exceeded 80%, validating that the sample size was adequate.

**Inclusion and exclusion criteria**

The inclusion and exclusion criteria for experimental animals were pre-established in the study protocol prior to the commencement of the experiments. Animals were included only if they: (1) successfully underwent the designated surgical procedure or treatment, (2) remained in good general health throughout the study period, and (3) had complete data records. Animals were excluded from the final analysis if they: (1) died prematurely due to surgical complications or anesthesia overdose (unrelated to the experimental treatment), (2) developed severe unexpected adverse effects or infections that compromised the welfare of the animal or the integrity of the experimental endpoint.

Inclusion and exclusion criteria for cell-based assays were defined prior to data collection. Data points from cell culture experiments were included only if they: (1) were derived from experiments with confirmed absence of mycoplasma contamination, (2) had enough technical replicates, and (3) passed quality control checks (adequate cell viability in control groups). Entire experimental runs were excluded from analysis if: (1) a technical failure occurred, or (2) the positive and negative controls failed to yield the expected results, indicating an invalid assay.

**Blinding procedures**

Investigators were blinded to group allocation during both the experimental procedures and outcome assessment. All samples/animals were coded by an independent researcher, and the code was not broken until completion of all data analyses.

**Reference**

1. Peng X, Sun B, Tang C, Shi C, Xie X, Wang X*, et al.* HMOX1-LDHB interaction promotes ferroptosis by inducing mitochondrial dysfunction in foamy macrophages during advanced atherosclerosis. *Dev Cell* 2024, **60**(7)**:** 1070-1086.

2. Lu W, Guo Y, Liu H, Zhang T, Zhang M, Li X*, et al.* The Inhibition of Fibrosis and Inflammation in Obstructive Kidney Injury via the miR-122-5p/SOX2 Axis Using USC-Exos. *Biomater Res* 2024, **28:** 0013.

**Supplementary Figures and Figure Legends**


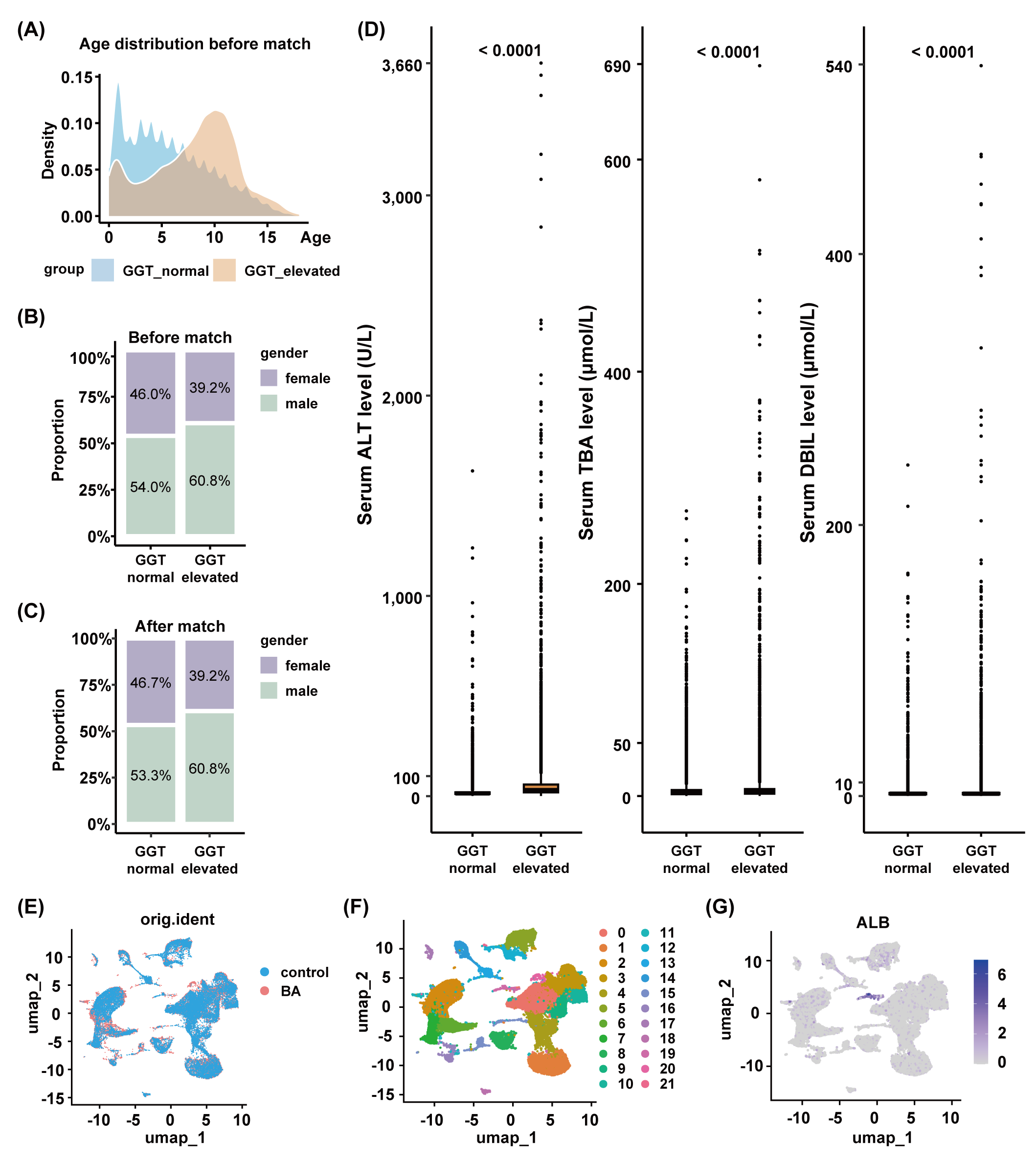


**Supplementary Figure 1. Elevated levels of serum copper and cholestatic bio-markers, and decreased FDX1 expression of hepatocytes in children with cholestasis, related to Figure 1**

(A) Density plot of age distribution between groups before matched for age and gender distribution. (B and C) Proportional stacked column charts of gender composition before and after matching for age and gender distribution. (D) Full-range boxplots displaying serum levels of ALT, TBA, and DBIL between GGT-normal group and GGT-evaluated group; see Figure 1C. (E, F, and G) UMAP plots illustrating clusters from scRNA-seq of BA livers and matched control livers without cholestasis (GEO: GSE176189; *n =* 3). Sample origin (E), cluster distribution (F), and ALB gene expression (G) of all clusters. Data are median (IQR). Mann-Whitney test (D).


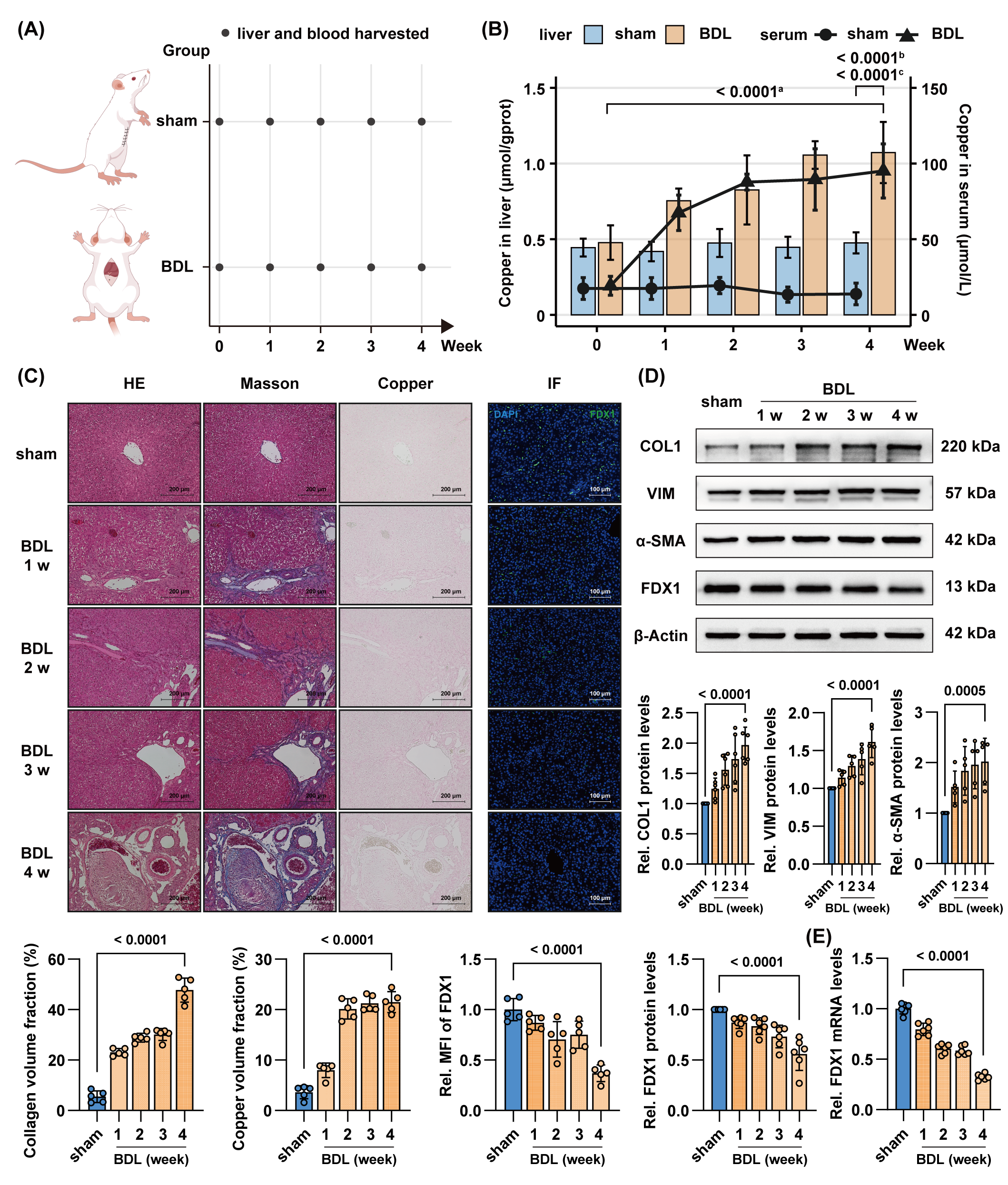


**Supplementary Figure 2. Chronic obstructive cholestasis induces dysregulated copper homeostasis with concomitant liver fibrosis and cuproptotic changes in rats, related to Figure 2**

(A) Schematic diagram of animal experiments. Liver and blood of rats were harvested at 0, 1, 2, 3, and 4 weeks post-operation. Liver tissue of sham group in panels B, C, and D was harvested on week 4. (B) Dynamic changes in serum and liver copper levels (*n =* 6). (C) Representative images of serial liver sections stained with H&E, Masson’s trichrome for collagen deposition (blue), dithiooxamide for copper salt deposits (green granules, *n =* 5), and representative images of immunoﬂuorescence of FDX1 (green) in liver tissue of rats (*n =* 5). Scale bar = 200 μm. Bottom: fold change in collagen volume fraction, copper volume fraction, and MFI of FDX1 relative to sham-week 4 group. (D) Western blot of COL1, VIM, α-SMA, and FDX1 in liver tissue of rats (*n =* 5 – 6). Bottom: fold change in COL1, VIM, α-SMA, and FDX1 expression relative to sham-week 4 group. (E) RT-qPCR of FDX1 in liver tissue of rats (*n =* 6). Fold change in FDX1 expression relative to sham-week 4 group. Data are mean ± SD. Two-way ANOVA with Tukey’s correction (B), one-way ANOVA with Šídák’s correction (C, D, and E). ^a^ Comparing serum values between BDL-week 0 and BDL-week 4 groups. ^b^ Comparing serum values between sham and BDL groups on week 4. ^c^ Comparing liver values between sham and BDL groups on week 4.


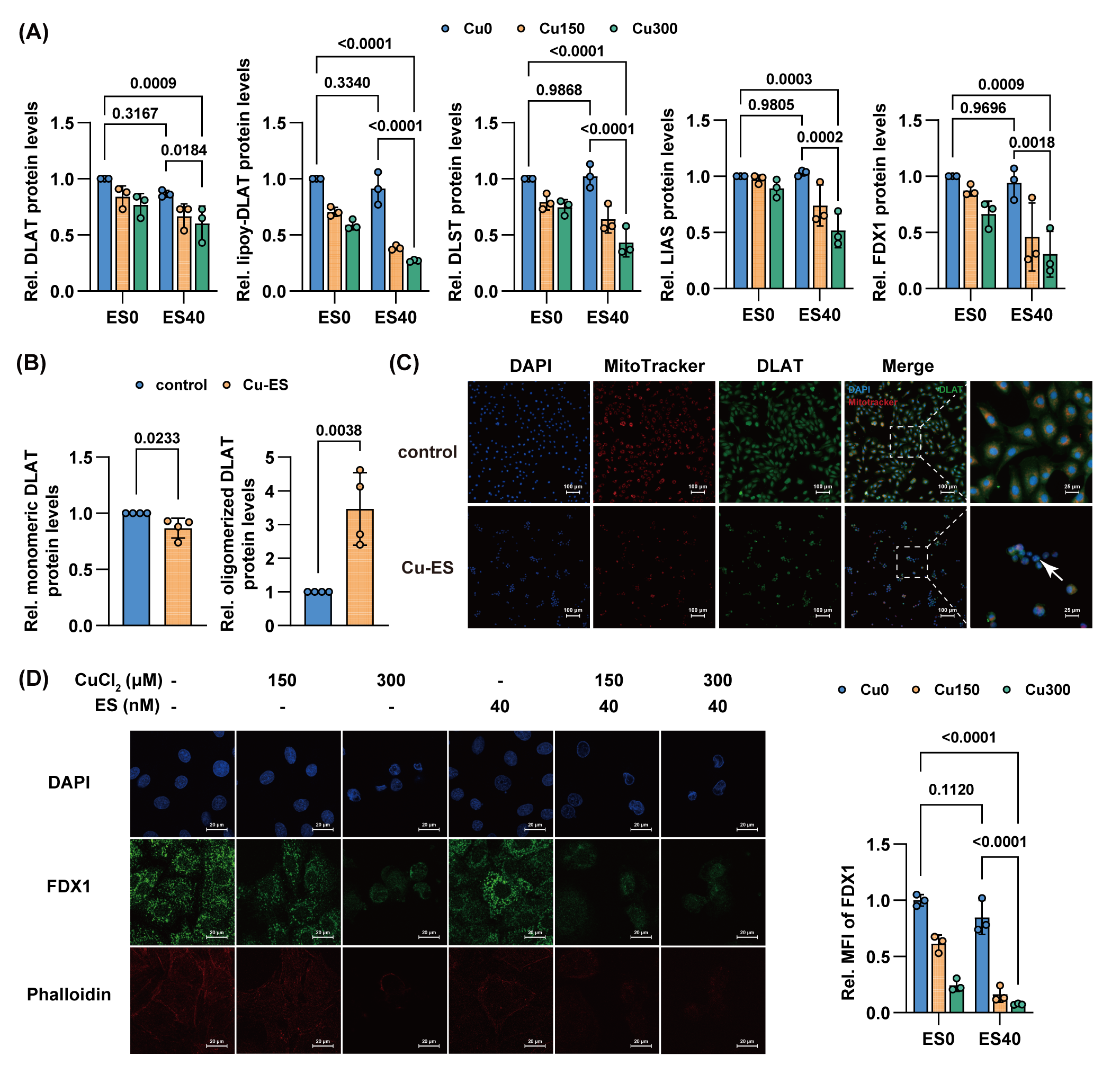


**Supplementary Figure 3. CuCl₂ and copper ionophores induce cuproptosis in rat hepatocytes, related to Figure 4**

(A) Comparative analysis of DLAT (non-lipoylated/lipoylated), DLST, LIAS, and FDX1 protein levels across experimental groups, normalized to the Cu0-ES0 group (see Figure 4D). (B) Comparative analysis of DLAT (monomeric form and oligomerized form) protein levels between groups, normalized to the control group (see Figure 4E). (C) Fluorescence images of separate and merged channels for DAPI (blue), MitoTracker (red), and DLAT (green) (*n* = 326 – 616 cells); related to Figure 4F. (D) Confocal fluorescence images of separate channels for DAPI (blue), Rhodamine phalloidin (red), and FDX1 (green); see Figure 4G. Right: fold change in FDX1 MFI relative to the Cu0-ES0 group. Data are mean ± SD. Two-way ANOVA with Šídák’s correction (A and D) and unpaired Student’s *t* test (B).


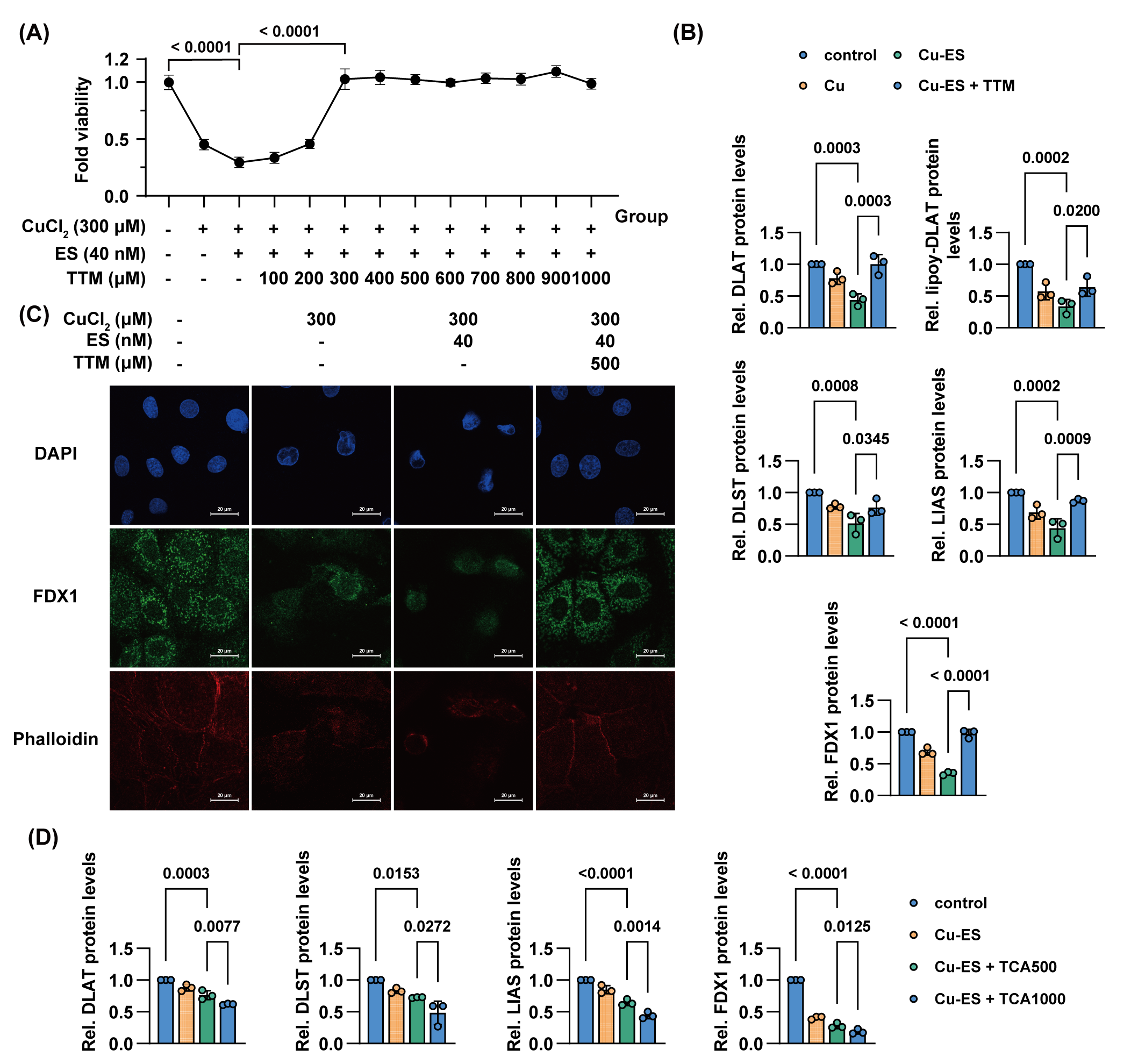


**Supplementary Figure 4. Copper overload induces distinct copper-dependent cell death and TCA further exacerbates cuproptosis via enhanced copper accumulation, related to Figure 5**

(A) Fold cell viability in BRL 3A cells post 24-h CuCl₂ (300 μM)/ES (40 nM) treatment, in combination with TTM in concentration gradients (0 – 1000 μM), relative to control group (*n =* 3). (B) Comparative analysis of DLAT (non-lipoylated/lipoylated), DLST, LIAS, and FDX1 protein levels across experimental groups, normalized to control group (see Figure 5C). (C) Confocal fluorescence images of separate channels for DAPI (blue), Rhodamine phalloidin (red), and FDX1 (green); see Figure 5D. (D) Comparative analysis of DLAT, DLST, LIAS, and FDX1 protein levels across experimental groups, normalized to control group (see Figure 5F). Data are mean ± SD. One-way ANOVA with Šídák’s correction (A, B and D).


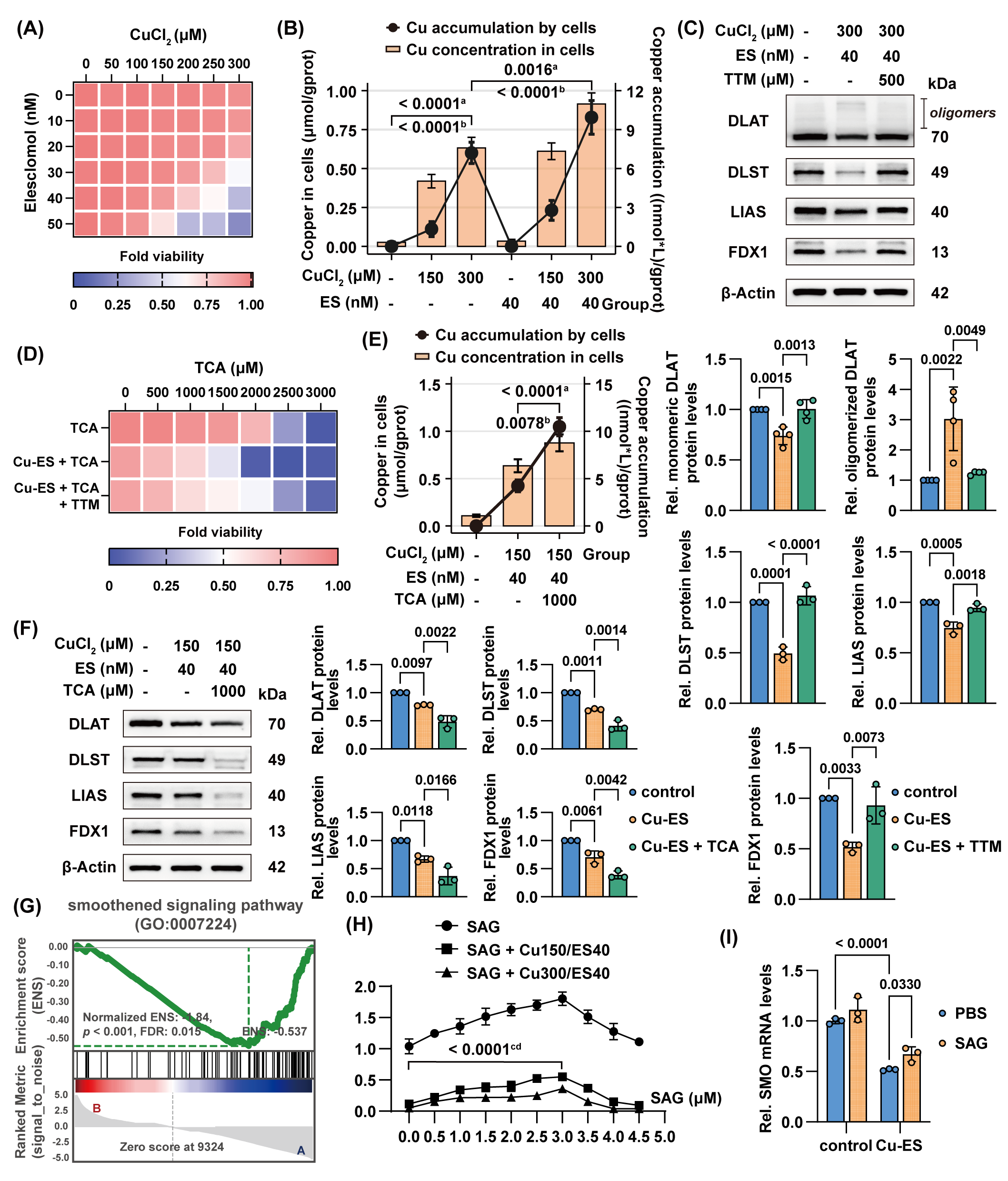


**Supplementary Figure 5. Phylogenetic conservation of copper overload-induced cuproptosis and TCA-mediated potentiation in mouse hepatocytes**

(A) Heatmap of CCK-8 assay for cell viability after 24-h CuCl₂ (0 – 300 μM) and ES (0 – 50 nM) treatment, relative to control group (*n =* 3). (B) Intracellular copper quantification and copper accumulation quantification after AML12 cells were treated with CuCl₂ (0, 150, and 300 μM) and ES (0 and 40 nM) for 24 h (*n =* 3). (C) Western blot of curproptosis-related proteins after AML12 cells were treated with CuCl₂ (300 μM) and ES (40 nM) in combination with TTM (500 μM) for 24 h (*n =* 3). Bottom: fold change in DLAT (monomeric form and oligomerized form), DLST, LIAS, and FDX1 expression relative to control group. (D) Heatmap of CCK-8 assay for cell viability after 24-h CuCl₂ (150 μM)/ES (40 nM)/TTM (500 μM) in combination with TCA (0 – 3000 μM) treatment, relative to control group (*n =* 3). (E and F) AML12 cells were treated with CuCl₂ (150 μM) and ES (40 nM) in combination with TCA (1,000 μM) for 24 h. Intracellular copper quantification and copper accumulation quantification (E; *n =* 3), and western blot of curproptosis-related proteins (F; *n =* 3). Right: fold change in DLAT, DLST, LIAS, and FDX1 expression relative to control group. (G) GSEA of DEGs (group B versus A; |log2 FC| > 1 and *q* value < 0.05) for smoothened signaling pathway from AML12 cells RNA-seq data; see Figure 6A–E. (H) AML12 cells were treated with CuCl₂ (150/300 μM) and ES (40 nM) in combination with SAG (0 – 4.5 μM) for 24 h. Line plots of CCK-8 assay for cell viability, relative to control group (*n =* 3). (I) RT-qPCR of SMO after AML12 cells were treated with CuCl₂ (150 μM) and ES (40 nM) in combination with SAG (3.0 μM) for 24 h, normalized to the control group (*n =* 3). Data are mean ± SD. Two-way ANOVA with Tukey’s correction (B), one-way ANOVA with Šídák’s correction (C, F and H), one-way ANOVA with Bonferroni’s correction (E), and two-way ANOVA with Šídák’s correction (I). ^a^ Comparing copper accumulation values between groups. ^b^ Comparing intracellular copper values between groups. ^c^ Comparing values in the SAG + Cu150/ES40 subgroup. ^d^ Comparing values in the SAG + Cu300/ES40 subgroup. Abbreviations: GSEA, gene set enrichment analysis.

**Supplementary Tables**

**Supplementary Table 1. De-identified laboratory records of pediatric patients (see file Supplementary Table 1.xlsx)**

**Supplementary Table 2. The siRNA targeting sequences for rat FDX1**

| **Product serial number** | **Product name** | **Targeting sequences** |
| --- | --- | --- |
| siG2204060203294896 | si-r-FDX1_001 | CTCAGAAGATAAGGTAACA |
| siG2204060203295996 | si-r-FDX1_002 | GAGGACCATATATATGAGA |
| siG2204060203297016 | si-r-FDX1_003 | CTAGATGTTGTGATTGAGA |

**Supplementary Table 3. Antibodies for western blot and immunostaining**

| **Antigens** | **Host** | **Company, Catalog number** |
| --- | --- | --- |
| FDX1 | Rabbit | Abclonal, A20895 |
| DLAT | Rabbit | Abclonal, A8814 |
| DLAT | Rabbit | Abclonal, A14530 |
| DLST | Rabbit | Abclonal, A6901 |
| LIAS | Rabbit | Proteintech, 11577-1-AP |
| Lipoic Acid | Rabbit | Abcam, ab58724 |
| α-SMA | Rabbit | Abclonal, A17910 |
| Vimentin | Rabbit | Abclonal, A11952 |
| Collagen 1 | Rabbit | Cell Signaling Technology, 72026 |
| SMO | Rabbit | Abclonal, A3274 |
| β-Actin | Mouse | Abclonal, AC004 |
| Rabbit IgG (HRP-conjugated) | Goat | Abclonal, AS014 |
| Mouse IgG (HRP-conjugated) | Goat | Abclonal, AS062 |
| Rabbit IgG (FITC-conjugated) | Goat | Abclonal, AS011 |

**Supplementary Table 4. The sequences of primers for RT-qPCR**

| **Gene** | **Gene ID** | **Species** | **Sequences (5'-3')** |
| --- | --- | --- | --- |
| *Actb* | 81822 | *Rattus norvegicus* | F: 5'-GAGGGTTACGCGCTCCC-3' |
|  |  |  | R: 5'-TAATGTCACGCACGATTTCCC-3' |
| *FDX1* | 29189 | *Rattus norvegicus* | F: 5'-AACGCTAACGACCAAGGGG-3' |
|  |  |  | R: 5'-CTCACACGCACCAAATCCATC-3' |
| *DLAT* | 81654 | *Rattus norvegicus* | F: 5'-ACCAAAGGGAAGGGTGTTCG-3' |
|  |  |  | R: 5'-AGGAGTTGGTGCCACTCTTG-3' |
| *LIAS* | 305348 | *Rattus norvegicus* | F: 5'-ACTACACTGCAAGTGGCCC-3' |
|  |  |  | R: 5'-TCTTTTAGCCACTAGATTTTTCAGG-3' |
| *Smo* | 25273 | *Rattus norvegicus* | F: 5'-ATGCCTTGATGGCTGGAGTA-3' |
|  |  |  | R: 5'-GCAGGTGGAAATAGGATGTCTT-3' |
| *Gli1* | 140589 | *Rattus norvegicus* | F: 5'-GCTCGCTCCGCAAACAT-3' |
|  |  |  | R: 5'-TCTCGCTTGGGCTCCACT-3' |
| *Gli2* | 304729 | *Rattus norvegicus* | F: 5'-CCATAAGCGGAGCAAGGTC-3' |
|  |  |  | R: 5'-CAGTGGCAGTTGGTCTCGTAG-3' |
| *Smo* | 319757 | *Mus musculus* | F: 5'-GAGCGTAGCTTCCGGGACTA-3' |
|  |  |  | R: 5'-CTGGGCCGATTCTTGATCTCA-3' |

**Original Western Blots**


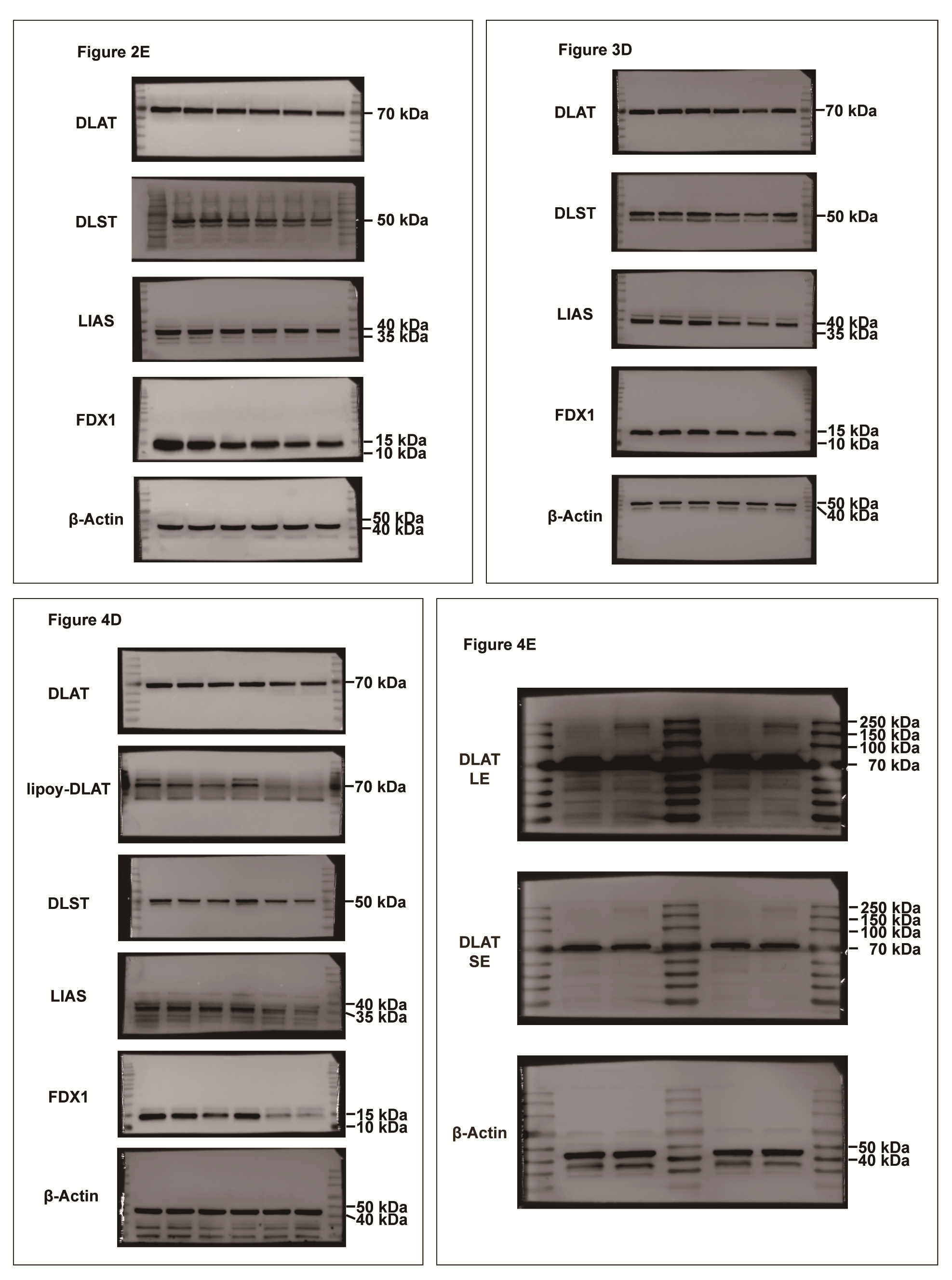


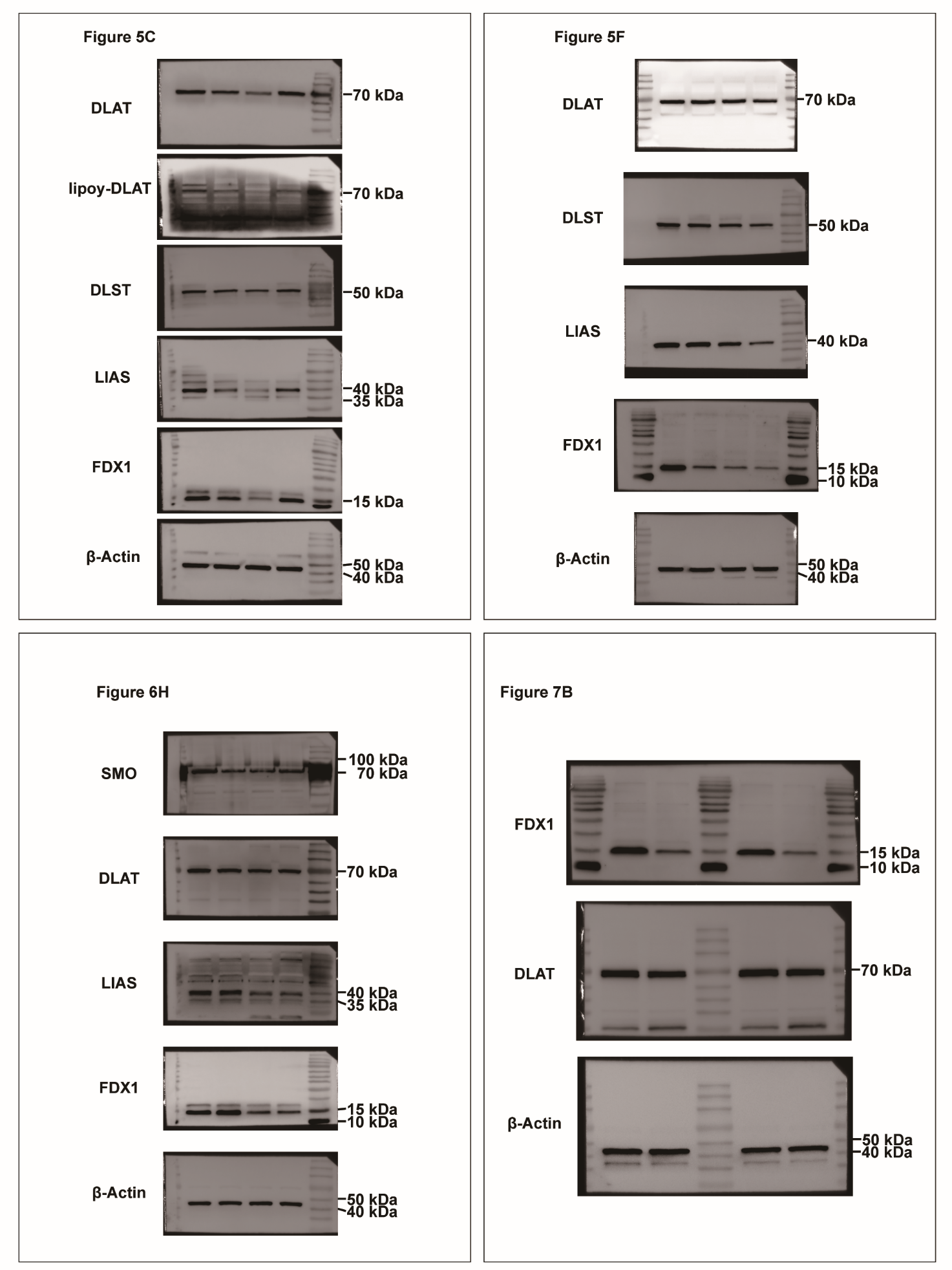


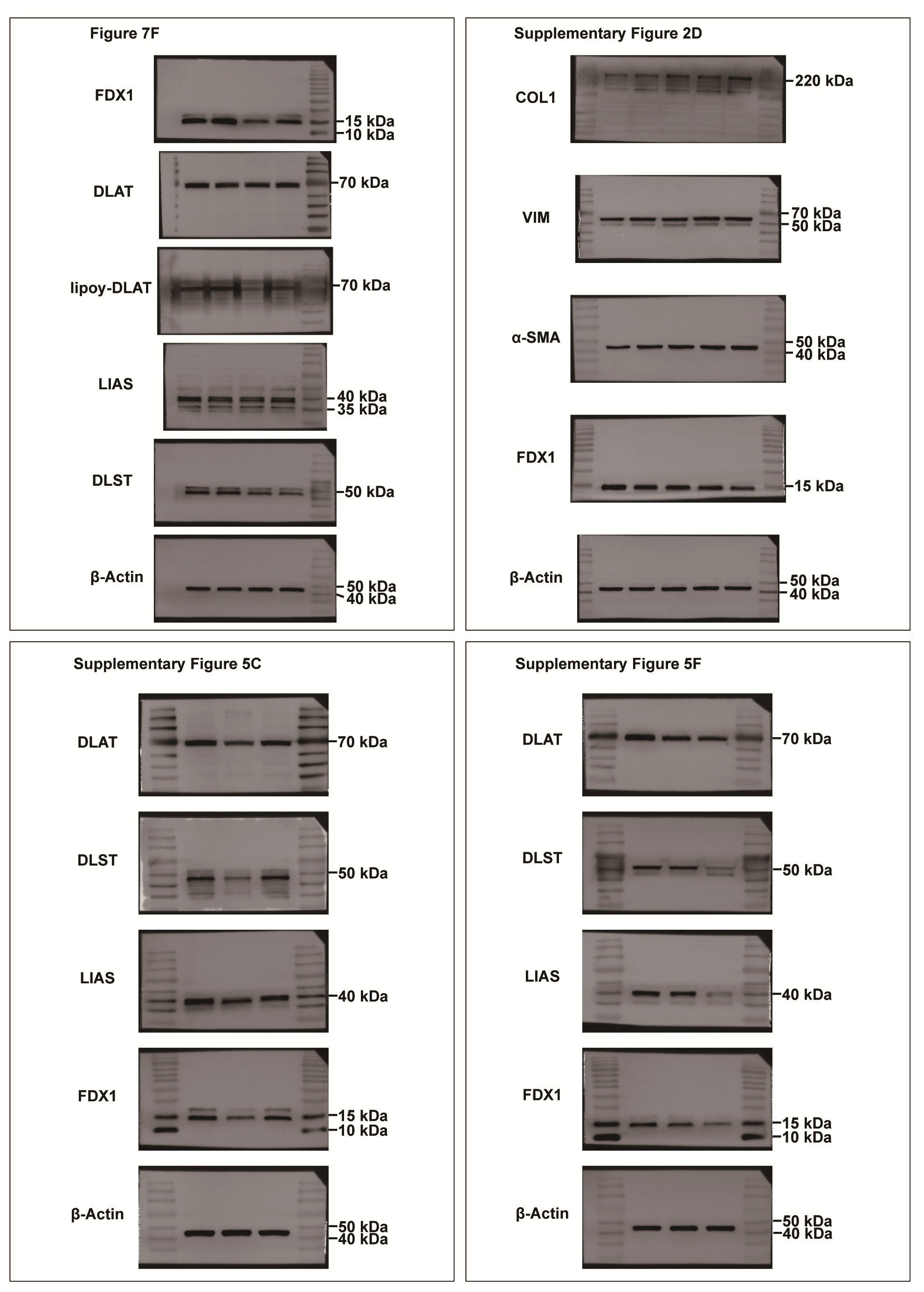

Supplement: Supplementary file 1 — Supplementary information [file 41420_2025_2861_MOESM1_ESM.docx]
